# Natural Variation of COLD and CATECHINS REGULATOR 1 Coordinately Fine‐Tunes Cold Tolerance and Tea Quality in Tea Plants

**DOI:** 10.1002/advs.76225

**Published:** 2026-06-22

**Authors:** Yanli Wang, Wei Tong, Qiong Wu, Didi Wu, Yanrui Zhang, Qijuan Gao, Fangdong Li, Zhaoliang Zhang, Wenjie Wang, Chuankui Song, Enhua Xia

**Affiliations:** ^1^ State Key Laboratory of Tea Plant Germplasm Innovation and Resource Utilization Anhui Agricultural University Hefei China; ^2^ Tea Research Institute Anhui Academy of Agricultural Sciences Hefei China

**Keywords:** Cold tolerance, *CsCCR1*, Catechins, Natural variation

## Abstract

Cold tolerance and tea quality are essential for profitable tea cultivation, yet the genetic interplay between them remains elusive. Here, we systematically evaluated cold tolerance and quantified 12 key tea quality‐related metabolites over two consecutive years of 108 geographically diverse tea accessions in China. Multi‐trait genome‐wide association mapping identified a central hub regulator, COLD AND CATECHINS REGULATOR 1 (CCR1), significantly associated with cold tolerance and catechins biosynthesis in tea plants. Functional analysis demonstrated that *CsCCR1* positively regulates cold tolerance and catechins accumulation in tea plants, and that a missense variation (A‐to‐C) in *CsCCR1* significantly enhances cold tolerance and catechins accumulation. Exogenous application of catechins (EGCG/EGC/EC) could also significantly alleviate cold‐induced oxidative damage through ROS scavenging. We demonstrated that *CsCCR1*, activated by upstream regulators CsLUX and CsKUA1, directly activates catechin biosynthetic genes (*CsCHS1*, *CsFLS1*, *CsSCPL4*, *CsSCPL5*) and a novel cold‐responsive gene *CsELIP1*. Furthermore, CsCCR1 physically interacts with CsCBF1/3 to form a transcriptional activation complex that synergistically amplifies expression of these downstream targets, thereby simultaneously modulating cold tolerance and catechins biosynthesis. Our findings underscore the importance of *CsCCR1* in linking cold tolerance with quality formation of tea plants, offering targets for breeding elite tea cultivars with superior cold resistance and quality.

## Graphical abstract

Multi‐trait genome‐wide association mapping identifies a central hub regulator, COLD AND CATECHINS REGULATOR 1 (CCR1), and its excellent natural allele variation, coordinately enhancing cold tolerance and promoting catechins biosyntheis. CsCCR1 interacts with CsCBF1/3 and is transcriptionally activated by CsLUX and CsKUA1 to promote catechins accumulation, thereby reinforcing the ROS scavenging capacity to maintain cellular homeostasis under cold stress. The findings underscore the importance of *CsCCR1* in linking cold tolerance with quality formation of tea plants, offering targets for breeding elite tea cultivars with superior cold resistance and quality.

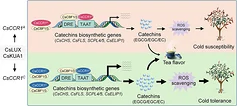

## 1 Introduction

Tea plant (*Camellia sinensis* L.) is an economically important perennial crop prized for its unique secondary metabolites, including catechins, theanine, and caffeine, which contributes to both its health benefits and commercial values [1]. As a plant species adapted to warm and humid environment, tea is particularly vulnerable to cold stress, which severely impairs tea growth, yield, and quality [2]. Cold‐induced damage not only limits the geographical distribution of tea cultivation but also leads to significant economic losses [3, 4], highlighting the urgent need to improve cold tolerance while maintaining or enhancing quality traits in tea plants.

The cold tolerance and quality traits of tea plants are genetically regulated by complex quantitative trait loci. Recent advances in plants have identified key genetic components involved in cold response and quality formation. Genome‐wide association studies (GWAS) have revealed several cold‐responsive genes, such as *COLD‐RESPONSIVE OPERATION LOCUS 1 (COOL1)* [5] and *CHILLING TOLERANCE DIVERGENCE 11 (COLD11)* [6], as well as quality‐related transcription factors like *WRKY19* and *NAC021* regulating aroma compounds of rice [7]. As is well known, the well‐conserved ICE‐CBF‐COR (inducer of CBF expression—C‐repeat binding factor—cold‐responsive genes) signaling pathway plays a central role in cold acclimation, with CsCBF1/2/3/5 serving as critical regulators in tea plants [8, 9, 10]. CsCBF1 has been reported to interact with plant‐specific ZINC FINGER‐HOMEODOMAIN/HOMEOBOX (ZHD/HB) transcription factor CsZHD9 to regulate its binding to the “TAAT” motif in the *CsMADS* promoter, which links the crosstalk between cold signaling and sprouting of tea buds [11, 12]. Notably, emerging evidence has suggested that extensive metabolic reprogramming is also triggered by cold stress, including both primary metabolites (e.g., amino acids, sugars) and specialized secondary metabolites like catechins, which are crucial for tea quality and stress adaptation [13, 14, 15].

Cumulative studies have revealed that catechins, the most abundant flavonoids in tea leaves, exhibit remarkable dual functionality. They not only determine the quality and health‐promoting properties of tea products, but also participate in abiotic stress responses [16, 17, 18, 19]. These compounds are mainly categorized into non‐galloylated (e.g., epicatechin (EC), epigallocatechin (EGC)) and galloylated (e.g., epigallocatechin gallate (EGCG), epicatechin gallate (ECG)) forms, synthesized through a coordinated pathway involving enzymes such as chalcone synthase (CHS), flavonol synthase (FLS), and serine carboxypeptidase‐like acyltransferases (SCPL) [19, 20, 21, 22]. Intriguingly, exogenous application of EGCG enhances cold tolerance in tea plants, and natural variation in catechin profiles correlates with cold sensitivity among tea varieties [14]. Furthermore, orthologs of catechin biosynthetic genes (e.g., *CHS*, *LAR*, *F3H*) have been shown to modulate cold tolerance in plants like kiwifruit, suggesting an evolutionarily conserved link between catechin metabolism and stress adaptation [23, 24, 25]. Despite these insights, the molecular mechanisms coordinating cold response and catechins accumulation in tea plants remain poorly understood. In particular, it is unclear whether specific regulatory nodes integrate these processes or if they are independently regulated. Revealing this knowledge gap could reveal novel breeding targets for developing tea cultivars with cold resilience and superior quality.

In this study, we employed an integrated multi‐omics methodology to unravel the genetic basis of cold tolerance and its relationship with catechins biosynthesis. Through systematic phenotyping of 108 diverse tea accessions across two consecutive winters and comprehensive metabolic profiling, we identified *HOMEOBOX 22* as a key regulator at the intersection of cold tolerance and catechins accumulation, which was further named as COLD AND CATECHINS REGULATOR 1 (CsCCR1). Functional characterization demonstrated that the CsLUX/CsKUA1‐*CsCCR1* module works through a dual regulatory mechanism, directly activating catechins biosynthetic genes (*CsCHS1*, *CsFLS1*, *CsSCPL4*, *CsSCPL5*) while also forming a CsCCR1‐CsCBF1/3 transcriptional complex to strongly induce expression of a novel cold‐responsive effector, *CsELIP1*. Our findings establish a *CsCCR1*‐centered regulatory module that synchronizes cold adaptation with quality metabolite biosynthesis, providing both fundamental insights into tea plant biology and practical strategies for cultivar improvement, which will also represent a significant advance in comprehending how economically important crops coordinate stress responses with quality trait formation.

## 2 Results

### 2.1 Identification and Characterization of Genes Synchronizing Cold Tolerance and Tea Quality in 108 Geographically and Phenotypically Diverse Tea Accessions

**FIGURE 1:**
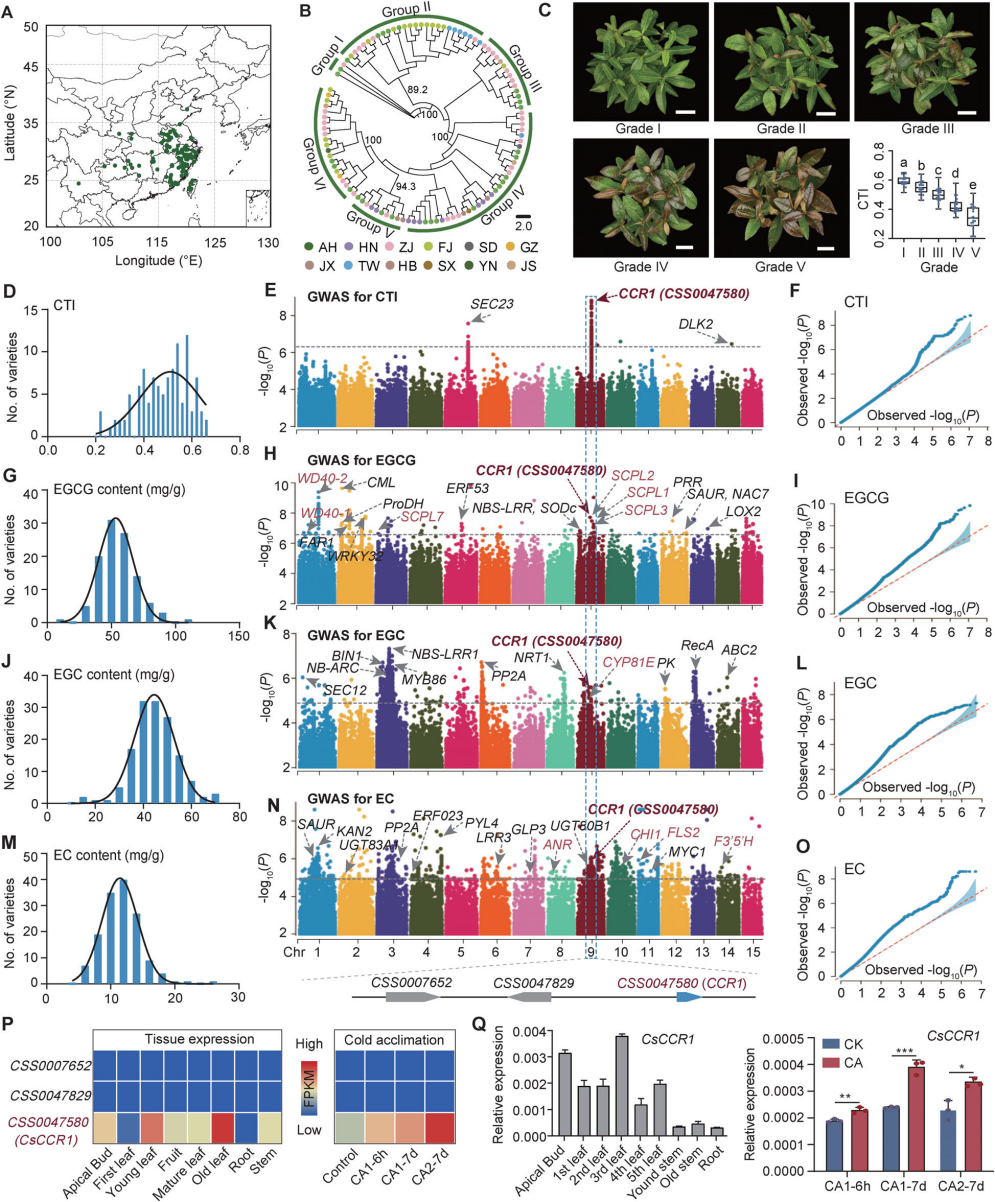
Identification of candidate genes associated with CTI and catechins. (A) Geographic distribution of 108 tea accessions across 12 tea‐producing provinces of China. The map of China was generated using an in‐house Python script (https://github.com/tw7649116/Tea_dispensable_genome) based on the open‐access geographic data (https://cloudcenter.tianditu.gov.cn/administrativeDivision). (B) Phylogenetic tree of 108 tea accessions constructed using genome‐wide SNPs, with tip colors corresponding to geographic origins. Province abbreviations: AH (Anhui), FJ (Fujian), ZJ (Zhejiang), GZ (Guizhou), HB (Hubei), HN (Hunan), JX (Jiangxi), JS (Jiangsu), SD (Shandong), SX (Shaanxi), TW (Taiwan), YN (Yunnan). (C) Representative phenotypes across five distinct cold tolerance grades. Scale bars = 5 cm. Data represent Mean±SD, n = 108. Multiple group comparisons were conducted via one‐way analysis of variance (one‐way ANOVA), followed by a two‐tailed Fisher's least significant difference (LSD) post hoc test. A value of *p* < 0.05 was considered statistically significant. (D) Frequency distribution of best linear unbiased estimates (BLUE) for CTI across two years. (E) Manhattan plot for CTI GWAS, with a horizontal line indicating the genome‐wide significance threshold (*p* < 4.50e‐7) based on Bonferroni correction. Arrows highlight candidate genes significantly associated with CTI. (F) Quantile–Quantile (Q–Q) plot for CTI GWAS with 1.05 of genomic inflation factor (λ). (G–O) Frequency distribution, GWAS, and QQ plot of EGCG, EGC, EC in the tea population. Known metabolic genes are denoted by red font. (P) FPKM (Fragments Per Kilobase of exon model per Million mapped fragments) of candidate genes (*CSS0007652*, *CSS0047829*, and *CSS0047580*) in representative tissue and cold stress. CA1‐6h: cold acclimated at 10°C for 6 h; CA1‐7d: 10/4°C (day/night) for 7 d; CA2‐7d: 4/0°C (day/night) for 7 d. (Q) RT‐qPCR analysis of *CsCCR1* in representative tissues and under cold stress. Data were denoted as Mean ± SD, n = 3. Statistical significance was determined by Student's *t*‐tests (^*^ *p* < 0.05, ^**^ *p* < 0.01, ^***^ *p* < 0.001).

We established an association panel of 108 diverse tea accessions extensively collected from 12 major tea‐producing regions across China, representing a broad geographic and genetic diversity of the collection. The panel was cultivated and maintained at the Tea Plant Germplasm Repository of the Tea Research Institute, Anhui Academy of Agricultural Sciences (Figure 1A and Data S1). Whole‐genome resequencing of this panel yields 6.84 Tb of high‐quality sequencing data, with an average depth of 22.97× and an average mapping rate of 98.88% to the reference genome (Figure 1A and Data S2). We identified a total of 13 383 259 high‐confidence single nucleotide polymorphisms (SNPs) distributed across 15 chromosomes (Figure S1B,C and Data S3). The high nucleotide diversity (*π* = 1.01E‐3) observed in these tea accessions confirms substantial genetic diversity of the population. Consistent with this finding, the phylogenetic and principal component analyses revealed no obvious stratification among accessions (Figure 1B and Figure S1D), supporting suitability of this panel for association mapping studies.

Cold tolerance of the 108 tea accessions was comprehensively evaluated using an integrated approach specifically adapted for perennial leaf‐harvested woody plants like tea [26, 27]. Unlike annual grain crops typically assessed by survival rate, the cold tolerance of tea plants was mainly determined through a well‐established protocol focusing on leaf damage under natural field conditions and physiological measurements under controlled laboratory environments. In line with this, we conducted field‐based observations of cold‐induced leaf injury alongside parallel laboratory qualification of key physiological parameters across 108 tea accessions over two consecutive years (Figure 1C; see Section 5). All measured physiological traits exhibited continuous variation with approximating normal distributions and showed significant interannual consistency (Figure S2A–N; *r* = 0.29; *p* = 8.63e‐3). According to the established methodologies [27, 28], we then subsequently developed a composite cold tolerance index (CTI) through CRITIC‐AHP weighting and membership function analysis to quantify overall cold hardiness of each accession. The resulting CTI values, consistent with field observations, exhibited a unimodal distribution peaking at 0.4–0.6, indicating substantial natural variation in cold tolerance among the accessions (Figure 1D).

In parallel, we quantified a total of 12 secondary metabolites associated with tea quality, including eight catechins (EC, ECG, EGC, EGCG, C, CG, GC, GCG), three methylxanthines (caffeine, theobromine, theophylline), and theanine, across the 108 tea accessions over two consecutive years. All metabolites showed normal distributions with substantial variation and demonstrated strong temporal consistency across years (*rr* = 0.51; *p* = 1.54e‐4; Figure S3). Among them, epigallocatechin gallate (EGCG) was the most abundant catechins, constituting 1.28% ∼ 12.51% of leaf dry weight (Data S4). Correlation analysis revealed the strongest positive association between EGCG and total catechins (*r* = 0.71), while the strongest negative correlation was observed between theophylline (THP) and EGCG (*r* = −0.32; Figure S4A,B). Notably, multivariate analysis revealed that CTI and total catechins accumulation shared similar distribution patterns (Figure S4C and Data S4), indicating a potential shared genetic regulation between cold tolerance and catechins biosynthesis in tea plants.

We then conducted a comprehensive genome‐wide association study (GWAS) based on the 13 383 259 high‐quality SNPs to dissect the genetic architecture of cold tolerance and secondary metabolites accumulation in tea plants (Figure S5A). For cold tolerance, the analysis identified four significant loci associated with CTI (*p* < 4.5e‐7), which corresponded to ten candidate genes (Figure 1E,F and Data S5). In parallel, GWAS of the twelve tea quality‐related metabolites, which showed diverse accumulation patterns across accessions (Figure 1G–M and Figures S3, S4), detected 7 544 significant SNPs, indicating the complex regulation of tea quality traits (Figure 1H–N; Figure S5B–I and Data S5). Notably, among the associated genes identified, 24 were functionally annotated in secondary metabolite biosynthesis, including key enzymes such as *CsSCPLs*, *CsCHI*, *CsANR*, *CsFLS*, and *CsANS*, as well as *MYB* transcription factors in tea plants.

A prominent and consistent association signal on chromosome 9 was identified for both cold tolerance and catechins accumulation across multiple years and statistical models (Figure 1E and Figure S6). This 150‐kb overlapping region exhibited pleiotropic effects, associated with CTI, EGCG, EGC, and EC accumulation (Figure 1E–N). Within this highly associated region, three candidate genes were annotated. Notably, one gene, *CSS0047580* (designated *CsCCR1*) was prioritized as the most promising candidate. Natural variations of *CsCCR1* in the coding region significantly altered the cold tolerance and quality traits of tea plants, as accessions carrying the CC allele (*CsCRR1^c^*) exhibited enhanced cold tolerance and elevated catechin content relative to those with the AA allele (*CsCRR1^A^*) (Figure S7A,B). Consistent with these genetic associations, *CsCCR1* showed predominant expression in tea buds and young leaves—tissues known to accumulate high levels of catechins, and was markedly upregulated during cold stress (Figure 1P–Q). Collectively, these findings strongly suggest that *CsCCR1* is likely a key genetic regulator potentially coordinating cold tolerance and quality‐related metabolite biosynthesis in tea plants.

### 2.2 *CsCCR1* Positively Regulates Cold Tolerance in Tea Plants

**FIGURE 2:**
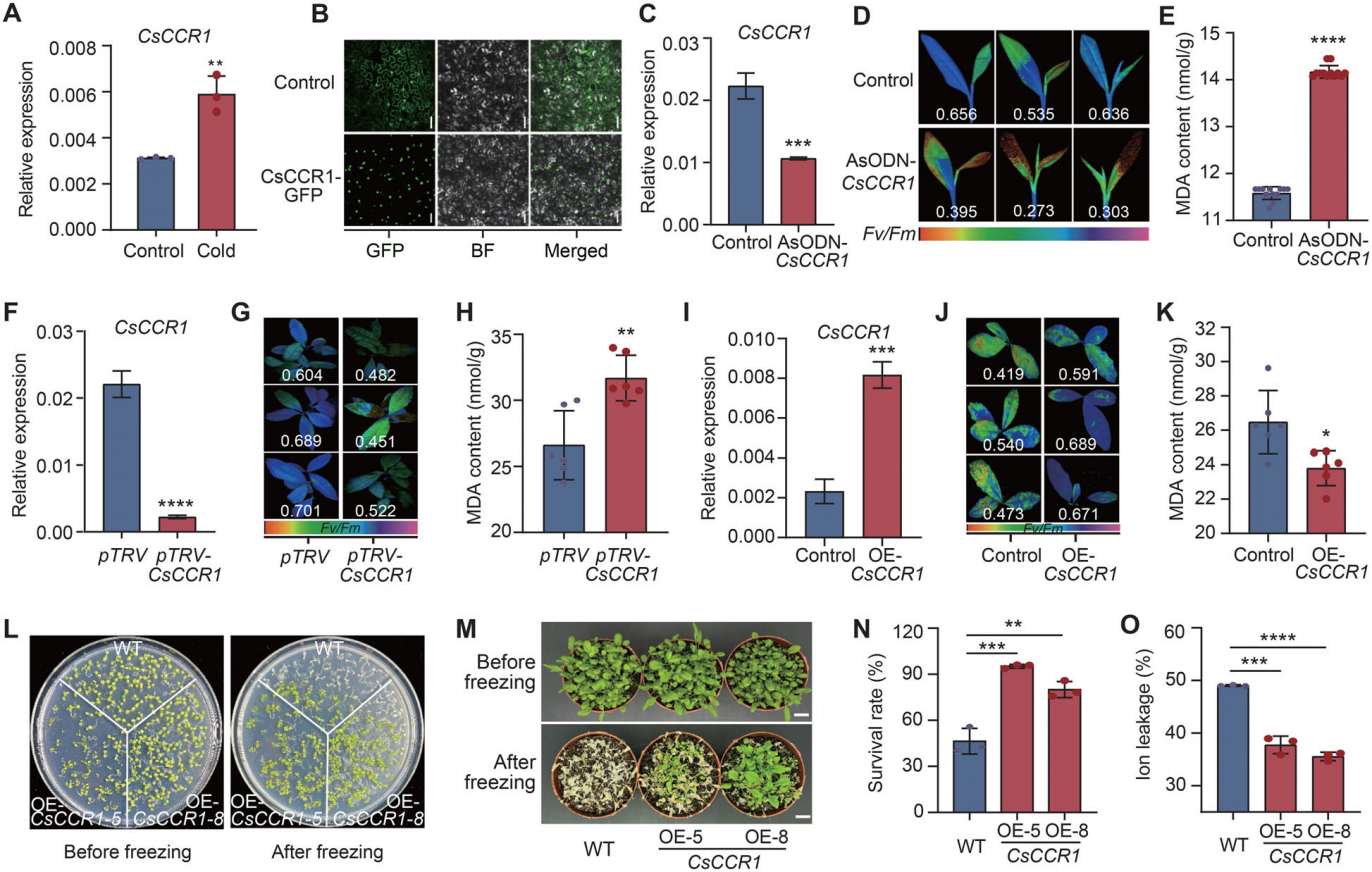
Functional validation of *CsCCR1* in cold tolerance of tea plants. (A) Expression profile of *CsCCR1* under cold stress. (B) Subcellular location of CsCCR1‐GFP fusion protein in tobacco epidermal cells, with green fluorescence protein (GFP) signal, bright field (BF), and merged image. Scale bars = 40 µm. (C) Expression of *CsCCR1* in one bud and two leaves treated with AsODN solution. (D) Chlorophyll fluorescence imaging in tender shoots under cold stress. The color bar represents damage severity via *Fv/Fm* value, with reduced damage from left to right. (E) MDA levels in *CsCCR1*‐suppressed and control tender shoots after freezing stress. (F) Virus‐induced gene silencing (VIGS) of *CsCCR1* in tea seedlings. (G) Chlorophyll fluorescence imaging of *CsCCR1‐*silenced seedlings after cold stress. (H) MDA levels of samples in the VIGS assay. (I–K) Functional characterization of *CsCCR1*‐overexpressed tea seedlings under cold stress. (L, M) Phenotypic comparison of wild‐type (WT) and *CsCCR1*‐overexpressing (OE*‐CsCCR1‐5* and OE*‐CsCCR1‐8*) *Arabidopsis* under cold stress (Scale bars = 2.0 cm). (N‐O) Survival rate and ion leakage of overexpressed samples. Data in the barplot were denoted as Mean ± SD, n = 3. Statistical significance was determined by Student's *t*‐tests (^*^ *p* < 0.05, ^**^ *p* < 0.01, ^***^ *p* < 0.001, ^****^ *p* < 0.0001).

To investigate the role of *CsCCR1* in cold tolerance, we first characterized its expression patterns and subcellular localization. Results showed that *CsCCR1* was highly induced by cold stress in tea plant leaves (Figure 2A). Subcellular localization assay revealed that CsCCR1 protein localized to the nucleus (Figure 2B and Figure S8A), in agreement with its predicted role as a transcriptional regulator. To functionally validate its role, we employed an antisense oligonucleotide (AsODN)‐mediated knockdown in tender shoots, which reduced *CsCCR1* expression by 52.25% (Figure 2C). Results showed that *CsCCR1*‐suppressing shoots exhibited significant cold sensitivity under cold stress, showing decreased chlorophyll fluorescence (*Fv/Fm*) and increased MDA levels relative to controls (Figure 2D,E). Consistently, virus‐induced gene silencing (VIGS) of *CsCCR1* in mature leaves also resulted in severe freezing damage, with reduced *Fv/Fm* and elevated MDA contents compared to controls (Figure 2F–H and Figure S8B).

We also transiently overexpressed *CsCCR1* in tea seedlings. This resulted in the enhanced cold tolerance, as demonstrated by elevated *Fv/Fm* and decreased MDA following freezing treatment (Figure 2I–K). To further substantiate this function across species, we generated 12 independent *CsCCR1*‐overexpressing *Arabidopsis* lines. RT‐qPCR analysis found that two lines (OE‐*CsCCR1*‐5 and OE‐*CsCCR1*‐8) had the highest expression levels among the transgenic lines (Figure S8C). These two lines displayed significantly improved cold tolerance compared to wild‐type (Figure 2L,M), as evidenced by both increased survival rates and reduced ion leakage in overexpressing lines (Figure 2N,O). Collectively, these results demonstrate that *CsCCR1* acts as a positive regulator of cold tolerance in tea plants.

### 2.3 *CsCCR1* Coordinates Cold‐induced Catechins Accumulation in Tea Plants

**FIGURE 3:**
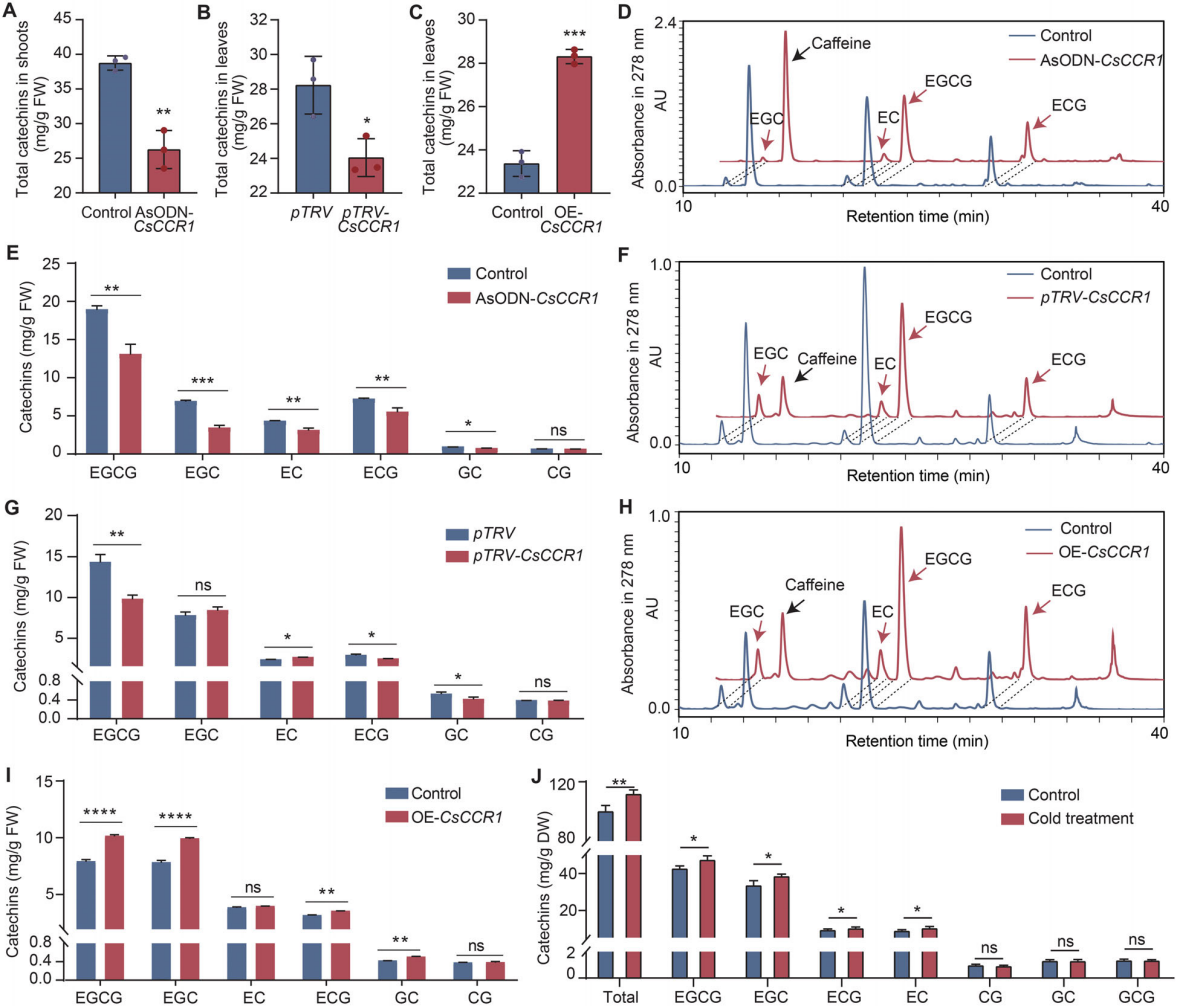
*CsCCR1*‐mediated catechins accumulation in response to cold. (A–C) Total catechins content in *CsCCR1*‐repressed, *CsCCR1*‐silenced and *CsCCR1*‐overexpressed samples, and their respective control tea seedlings. FW: fresh weight. (D) HPLC chromatographs of catechins in young leaves of *CsCCR1*‐repressed and control set. (E) Individual catechin compounds in *CsCCR1*‐repressed and control tea seedlings. (F,G) HPLC chromatographs and content of individual catechin compounds in *CsCCR1*‐silenced and control tea seedlings. (H,I) Individual catechin profiles in *CsCCR1*‐overexpressed and control tea seedlings. (J) Total and individual catechin contents in tea seedlings under cold stress. Retention time: 21.327‐21.538 for EGCG, 12.514‐12.700 for EGC, 29.088‐29.242 for ECG, and 20.058‐20.270 for EC. All data were denoted as Mean ± SD, n = 3. Statistical significance was determined by student's *t*‐tests (^*^ *p* < 0.05, ^**^ *p* < 0.01, ^***^ *p* < 0.001, ^****^ *p* < 0.0001). “ns” indicates no significance.

The significant GWAS associations between *CsCCR1* and EGCG (*p* = 2.21e‐7), EGC (*p* = 2.05e‐5), and EC (*p* = 4.46e‐5) also prompted us to further investigate the functional relevance of *CsCCR1* on catechins accumulation (Figure 1H–O). The results showed that *CsCCR1*‐silenced plants exhibited significant reductions in total catechins content, while overexpression lines showed significant increases of total catechins (Figure 3A–C). To quantify the individual catechins affected most, we performed high‐performance liquid chromatography (HPLC) analysis on *CsCCR1*‐silenced tea shoots using AsODN (Figure S8D,E). The results showed that suppression of *CsCCR1* resulted in reduced peak areas and significantly decreased levels of EGCG, ECG, EC, EGC, and GC (Figure 3D,E). Consistent with these findings, VIGS‐mediated silencing of *CsCCR1* also led to decreased accumulations of EGCG, ECG, and GC in tea leaves compared to controls (Figure 3F,G). Conversely, *CsCCR1*‐overexpressing tea leaves exhibited significantly elevated catechins levels, particularly EGCG, EGC, ECG, and GC, relative to the control group (Figure 3H,I).

We also conducted targeted metabolomic profiling of tea plants under cold stress. The results found that 90.91% of detected catechins were upregulated by cold stress among all differentially accumulated flavonoids metabolites (Figure S8F,G and Data S6). Specific quantification further confirmed that the total catechins (11.81%), EGCG (10.84%), EGC (14.55%), and EC (15.25%) were significantly increased after cold treatment (Figure 3J). These results jointly indicate that *CsCCR1* mediates catechins accumulation in response to cold stress in tea plants.

### 2.4 *CsCCR1* Directly Binds to the Promoters of Five Key Catechins Biosynthetic Genes *CsCHS1/CsFLS1/CsSCPL4/CsSCPL5/CsELIP1* and Activates Their Expression

**FIGURE 4:**
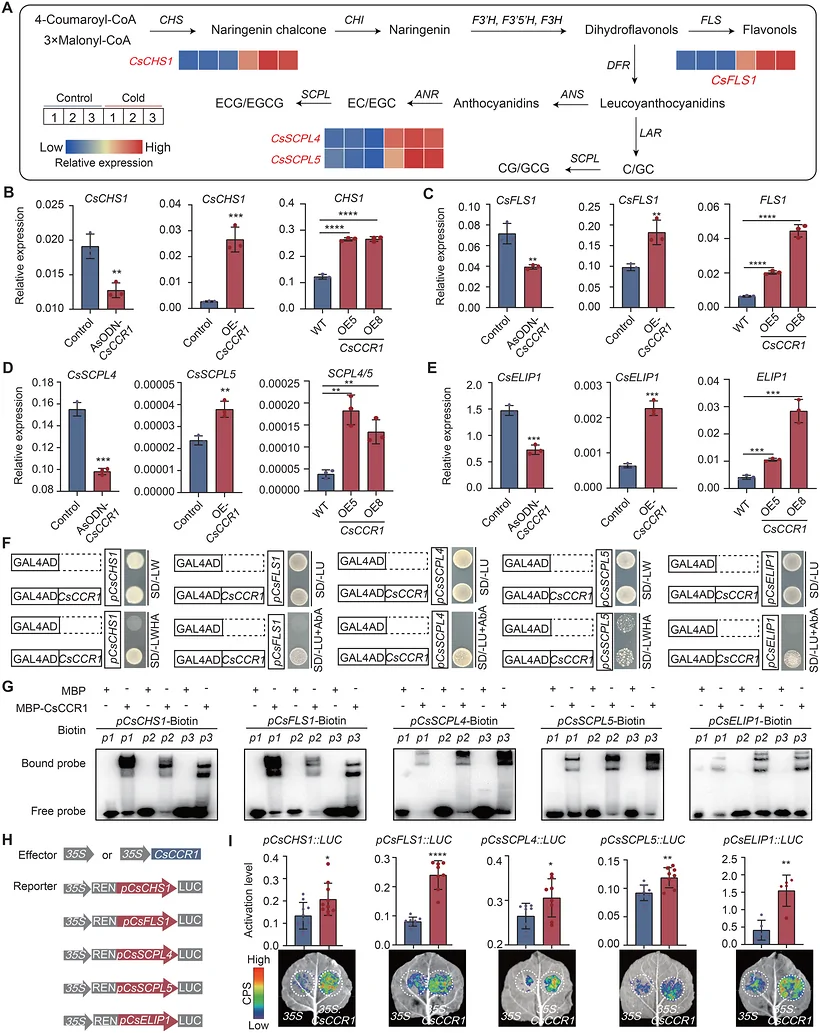
CsCCR1 directly activates the expression of catechins biosynthetic genes *CsCHS1*, *CsFLS1*, *CsSCPL4*, *CsSCPL5*, and *CsELIP1*. (A) Relative expression levels of *CsCHS1*, *CsFLS1*, *CsSCPL4*, and *CsSCPL5* involved in catechins biosynthetic pathway under cold stress. (B) *CsCHS1* expression in *CsCCR1*‐silenced and *CsCCR1*‐overexpressed (OE) tea plants compared to controls, and *AtCHS1* expression in *CsCCR1*‐OE *Arabidopsis* versus wild‐type (WT). (C–E) *CsFLS1/CsSCPL4/ CsSCPL5/CsELIP1* expression in *CsCCR1*‐silenced and OE‐*CsCCR1* tea plants compared to controls, and *AtCHS1/FLS1/SCPL4/SCPL5/ELIP1* expression in *CsCCR1*‐OE *Arabidopsis* versus WT. (F) Y1H assay showing CsCCR1 binding to the *pCsCHS1*, *pCsFLS1*, *pCsSCPL4*, *pCsSCPL5*, and *pCsELIP1*. L: Leucine, W: Tryptophan, H: Histidine, A: Adenine, U: Uracil, AbA: Aureobasidin A. Concentration used to suppress auto‐activation: 200 ng/mL AbA for *pCsFLS1* and *pCsSCPL4*, 300 ng/mL AbA for *pCsELIP1*, and 50 mm 3AT for *pSCPL5*. (G) EMSA confirms the binding of CsCCR1 to three TAAT‐containing fragments in *pCsCHS1*, *pCsFLS1*, *pCsSCPL4*, *pCsSCPL5*, and *pCsELIP1*. (H) Schematic of effector and reporter constructs for Dual‐LUC assays. (I) CsCCR1 transactivates *CsCHS1*, *CsFLS1*, *CsSCPL4*, *CsSCPL5*, and *CsELIP1* promoters. Data were denoted as Mean ± SD, n = 3. Significance was determined by Student's *t*‐tests (^*^ *p* < 0.05, ^**^ *p* < 0.01, ^***^ *p* < 0.001, ^****^ *p* < 0.0001).

To elucidate the mechanism by which *CsCCR1* regulates catechin biosynthesis and cold tolerance in tea plants, we first systematically examined the expression patterns of all catechins‐related genes under cold stress and in *CsCCR1*‐transgenic tea seedlings. The results showed that the expression levels of four key catechins biosynthetic genes, including *CsCHS1*, *CsFLS1*, *CsSCPL4*, and *CsSCPL5*, were significantly induced by cold stress (Figure 4A, Figure S9A–D). Notably, we also identified a novel gene functionally annotated in flavonoid biosynthesis that exhibited strong upregulation under cold stress (Figure S9E). Phylogenetic and domain analyses identified it as early light‐induced protein 1 (CsELIP1), and our data showed that CsELIP1 positively regulates catechins biosynthesis of tea plants and cold tolerance of transgenic *Arabidopsis* (Figure S10). Expression analysis further revealed that *CsCHS1*, *CsFLS1*, *CsSCPL4*, *CsSCPL5*, and *CsELIP1* were downregulated in *CsCCR1*‐suppressed tea shoots, while their expression and that of their homologous were markedly upregulated in both *CsCCR1*‐overexpressing tea seedlings and transgenic *Arabidopsis* (Figure 4B–E). These consistent expression patterns suggested potential regulatory relationships between *CsCCR1* and these catechins‐related genes.

To determine whether CsCCR1 directly regulates these genes, we performed yeast one‐hybrid (Y1H) assays and found that CsCCR1 could directly bind to the promoters of the five genes (Figure 4F). For precise mapping of the binding sites, we designed three 38‐bp probes containing TAAT motif at 500‐bp intervals within each *CsCHS1*, *CsFLS1*, *CsSCPL4*, *CsSCPL5*, and *CsELIP1* promoter. Further electrophoretic mobility shift assays (EMSA) showed that CsCCR1 could bind to all three TAAT motifs in the promoters of these genes (Figure 4G). Furthermore, dual‐luciferase (LUC) reporter assays revealed that CsCCR1 can significantly activate the expression of *CsCHS1*, *CsFLS1*, *CsSCPL4*, *CsSCPL5*, and *CsELIP1*, but not other catechin biosynthetic genes compared to the empty vector controls (Figure 4H–I and Figure S11). These findings demonstrated that CsCCR1 serves as a direct transcriptional activator of *CsCHS1*, *CsFLS1*, *CsSCPL4*, *CsSCPL5* and *CsELIP1*, thereby positively regulating catechins biosynthesis pathway in response to cold stress in tea plants.

### 2.5 *CsCHS1/CsFLS1/CsSCPL4/CsSCPL5/CsELIP1* Positively Regulate Cold Tolerance and Catechins Biosynthesis in Tea Plants

To validate the functions of *CsCHS1*, *CsFLS1*, *CsSCPL4*, *CsSCPL5*, and *CsELIP1* in cold tolerance and catechins biosynthesis in tea plants, we first checked their expression patterns in representative tissues and under cold stress. Results showed that *CsCHS1*, *CsFLS1*, *CsSCPL4*, *CsSCPL5*, and *CsELIP1* were predominantly expressed in apical bud and young leaves, exhibiting consistent spatial patterns with catechins accumulation in tea plants [22] (Figure S12A–C). Under cold stress, these genes were markedly upregulated, with *CsCHS1* showing a 10.13‐fold increase, *CsFLS1* 4.20‐fold, *CsSCPL4* 4.99‐fold, *CsSCPL5* 1.72‐fold, and *CsELIP1* exhibiting the most pronounced induction (25.14‐fold) compared to controls (Figure S12B,C). Subcellular location showed that CsCHS1 and CsFLS1 are localized in the cytoplasm and nucleus, while CsELIP1 is localized in the chloroplast (Figure S12D).

**FIGURE 5:**
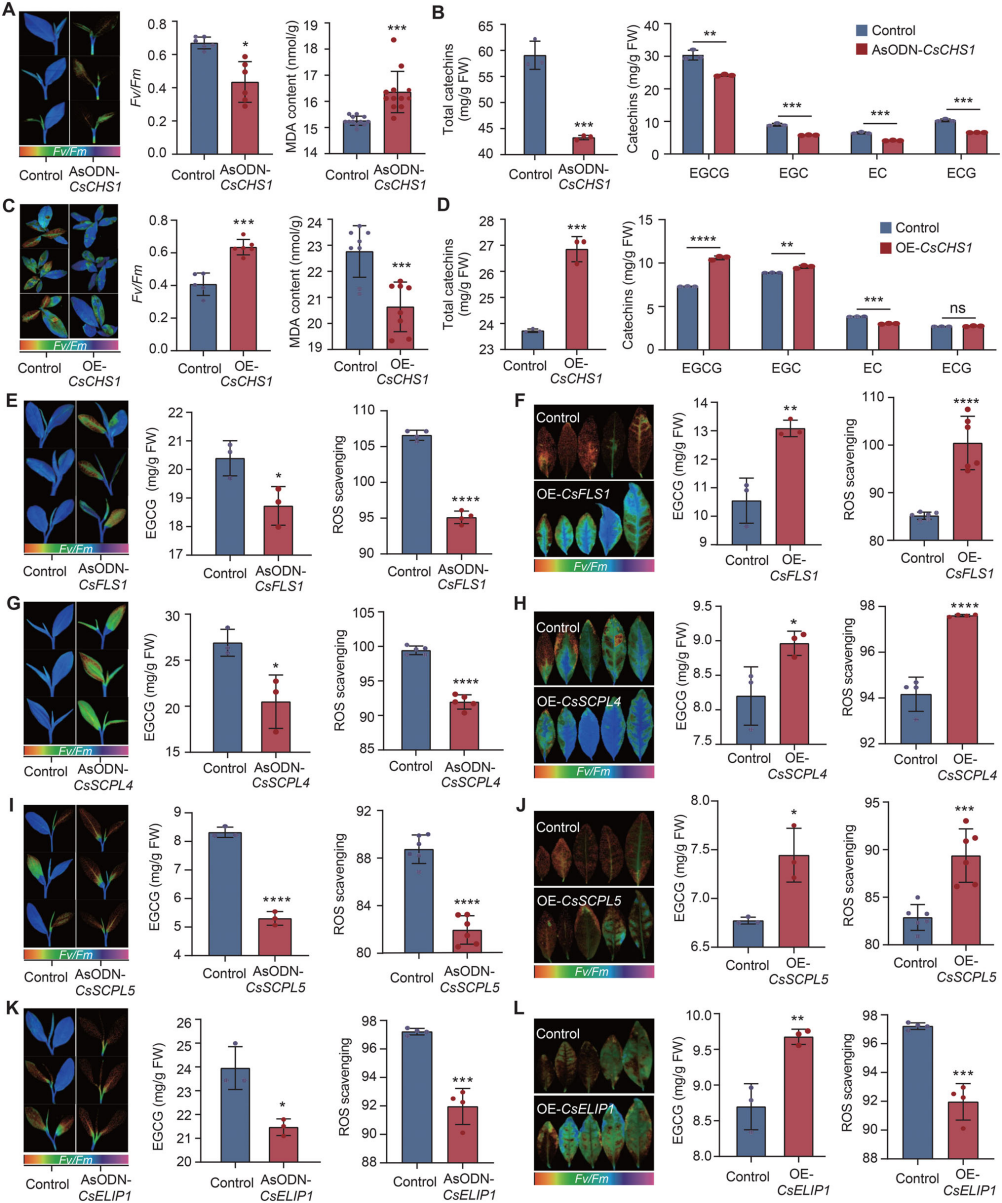
Functional characterization of *CsCHS1*, *CsFLS1*, *CsSCPL4*, *CsSCPL5*, and *CsELIP1* in cold tolerance and catechins biosynthesis in tea plants. (A) Freezing phenotype and physiological indicators of *CsCHS1*‐repressed and control tea shoots. (B) Total catechins accumulation and individual EGCG, EGC, EC, ECG content in *CsCHS1*‐repressed and control tea shoots. (C) Freezing tolerance assays and stress‐related physiological indices in *CsCHS1*‐overexpressing and control tea seedlings. (D) Catechins accumulation in *CsCHS1*‐overexpressing and control tea seedlings. (E) Freezing phenotype and EGCG accumulations of *CsFLS1*‐silenced tea shoots. (F) Freezing tolerance and EGCG accumulations in *CsFLS1*‐overexpressing seedlings. (G,H) Freezing phenotype and physiological alterations in *CsSCPL4*‐silenced and *CsSCPL4*‐overexpressing tea plants. (I,J) Freezing phenotype and physiological indicators of *CsSCPL5*‐silenced and *CsSCPL5*‐overexpressing tea seedlings. (K,L) Freezing phenotype and physiological indicators of *CsELIP1*‐silenced and *CsELIP1*‐overexpressing tea seedlings. Data were denoted as Mean ± SD, n = 3. Significance was determined by Student's *t*‐tests (^*^ *p* < 0.05, ^**^ *p* < 0.01, ^***^ *p* < 0.001, ^****^ *p* < 0.0001).

To characterize the roles of these genes in cold tolerance, we respectively inhibited and overexpressed their expression in tea seedlings (Figure S13). The results showed that *CsCHS1*‐inhibited tea seedlings were more sensitive to cold stress compared to controls (Figure 5A). Notably, *CsCHS1* suppression also led to significant reductions in total catechins and individual flavan‐3‐ols (EGCG, EGC, EC, and ECG) in young leaves (Figure 5B). Conversely, *35S*‐driven overexpression of *CsCHS1* enhanced cold tolerance in transgenic tea seedlings, concomitant with elevated EGCG and EGC accumulations relative to control tea seedlings (Figure 5C,D). These data indicate that *CsCHS1* could promote cold tolerance and catechins accumulation in tea plants.

Parallel investigations of *CsFLS1* also revealed its critical role in cold resilience of tea plants. Results showed that *CsFLS1*‐silenced tea shoots exhibited reduced cold tolerance with reduced EGCG accumulations and ROS acvenging ability compared to controls (Figure 5E). In contrast, *CsFLS1*‐overexpressing tea seedlings showed elevated cold resilience, with improved EGCG accumulations and ROS scavenging ability compared to control (Figure 5F). Similar functional patterns were also observed for *CsSCPL4*, *CsSCPL5* and *CsELIP1*. Overexpression of these genes conferred improved cold resistance, EGCG levels, and ROS scavenging ability in tea seedlings, while silencing their expression led to decreased cold tolerance, EGCG contents, and ROS scavenging ability compared to controls (Figure 5G–L and Figure S23A–C). Collectively, these findings demonstrate that *CsCHS1*, *CsFLS1*, *CsSCPL4*, *CsSCPL5*, and *CsELIP1* coordinately regulate cold tolerance and catechins biosynthesis, establishing a potential link between secondary metabolite accumulation and cold tolerance in tea plants.

### 2.6 CsCCR1 Physically Interacts With CsCBF1 and CsCBF3 to Enhance Transcription of Catechins Biosynthetic Genes Under Cold Stress of Tea Plants

**FIGURE 6:**
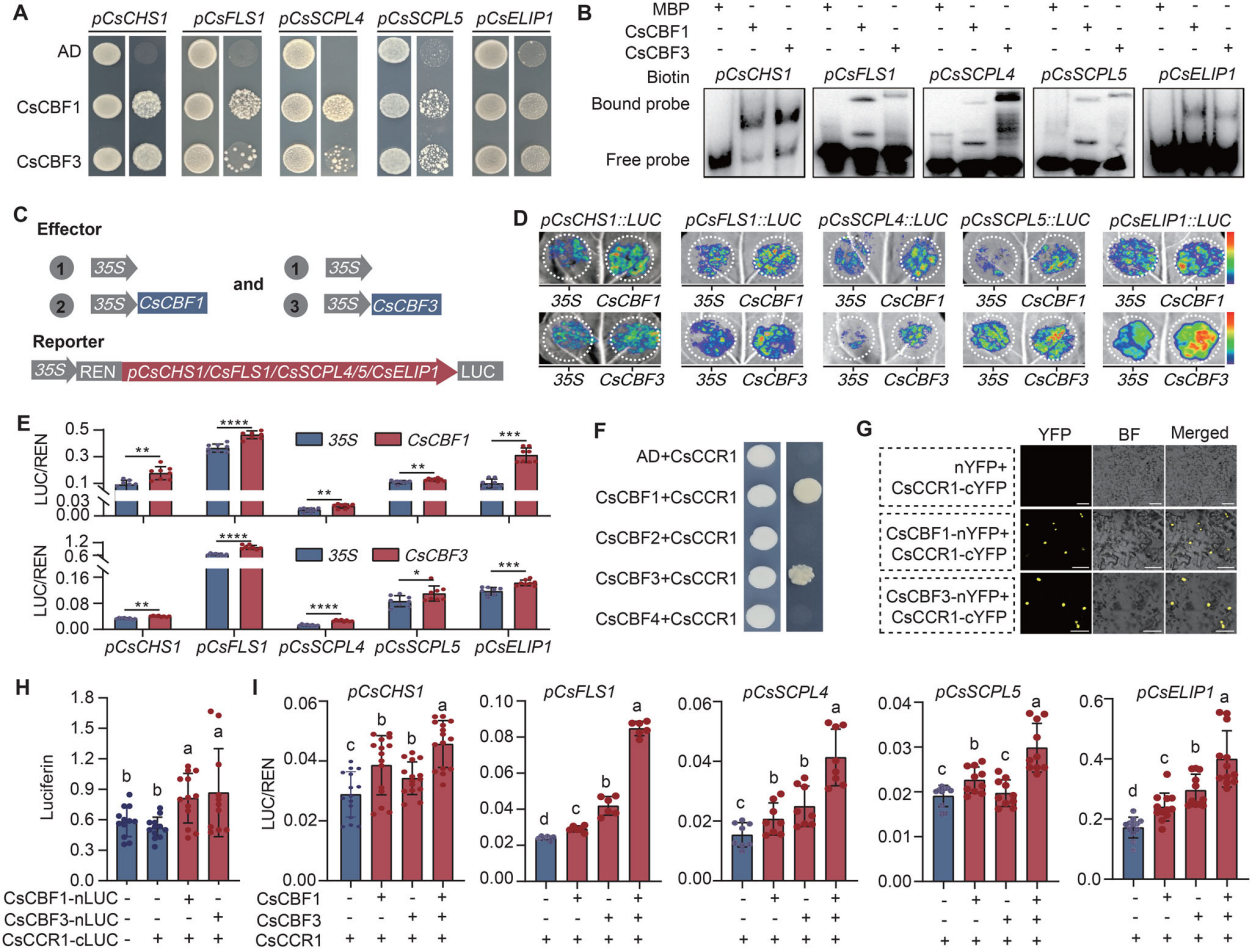
CsCBF1‐CsCCR1‐CsCBF3 regulatory complex synergistically activates the expression of catechins biosynthetic genes. (A) Y1H assays showing CsCBF1 and CsCBF3 binding to promoter of *CsCHS1*, *CsFLS1*, *CsSCPL4*, *CsSCPL5*, and *CsELIP1*. Left column (each panel): control group on yeast screening medium (‐Leu/‐Trp); right column: yeast growth on synthetic dropout medium (‐Leu/‐Trp/‐His). Concentration used to suppress auto‐activation: 25 mm 3AT for *pCsFLS1* and *pCsSCPL4*, 50 mm 3AT for *pSCPL5*, 300 ng/mL AbA for *pCsELIP1*. (B) EMSA showing MBP‐CsCCR1 binding to CAAT‐containing 38‐bp fragments from *pCsCHS1*, *pCsFLS1*, *pCsSCPL4*, *pCsSCPL5*, and *pCsELIP1*, respectively. (C) Schematic of effector and reporter constructs for Dual‐LUC assays. (D,E) Relative LUC/REN ratios showing CsCCR1‐mediated activation on target promoters. The color scale represents fluorescence intensity from low (bottom) to high (top). Significance was determined by Student's *t*‐tests (^*^ *p* < 0.05, ^**^ *p* < 0.01, ^***^ *p* < 0.001, ^****^ *p* < 0.0001). (F) Y2H assays in Y2H Gold strain confirming interaction of CsCCR1 with CsCBF1, and CsCBF3. (G) BiFC visualization of CsCCR1‐CsCBF1/CsCBF3 interactions in *N. benthamiana* nuclei (YFP signal). Scale bars = 40 µm. (H) LCI assay quantifying CsCCR1‐cLUC interactions with CsCBF1‐nLUC/CsCBF3‐nLUC compared to negative controls. (I) Synergistic activation of CsCCR1, CsCBF1, and CsCBF3 on target promoters (*pCsCHS1*, *pCsFLS1*, *pCsSCPL4*, *pCsSCPL5*, and *pCsELIP1*) versus individual effectors (normalized to Renilla luciferase). Data represent Mean ± SD of ≥ 3 biological replicates. Multiple group comparisons were conducted via one‐way analysis of variance (one‐way ANOVA), followed by a two‐tailed Fisher's least significant difference (LSD) post hoc test. Value of *p* < 0.05 was considered statistically significant.

It was widely accepted that C‐repeat binding factor (CBF) plays a central role in cold response in plants [2, 8]. Our analysis identified putative CBF‐binding dehydration‐responsive elements (DRE, CCGAC) in the promoters of *CsCHS1*, *CsFLS1*, *CsSCPL4*, *CsSCPL5*, and *CsELIP1*. The results led us to hypothesize that these genes may participate in CBF‐dependent cold stress response in tea plants (Figure S14). To test this, we first examined whether CsCBFs directly regulate these catechin biosynthetic genes. The results from Y1H and EMSA assays both showed that, among the four CBF members of tea plants, only CsCBF1 and CsCBF3 could directly bind to the DRE elements in the promoters of *CsCHS1*, *CsFLS1*, *CsSCPL4*, *CsSCPL5*, and *CsELIP1* (Figure 6A,B). Dual‐LUC assays further demonstrated that CsCBF1 and CsCBF3 could independently activate the transcription of these target genes (Figure 6C–E), indicating their direct regulatory roles in cold tolerance and catechin biosynthesis.

The convergent regulation of catechins‐related genes by CsCCR1 and CsCBF1/3 further prompted us to investigate the potential interactions among these proteins. Yeast two‐hybrid (Y2H) assays revealed that CsCCR1 specifically interacts with CsCBF1 and CsCBF3, but not with other CsCBF members (Figure 6F). This interaction was further confirmed by bimolecular fluorescence complementation (BiFC) assays, in which co‐expression of CsCCR1 with either CsCBF1 or CsCBF3 reconstituted yellow fluorescent protein (YFP) fluorescence specifically in the nucleus, consistent with the nuclear localization of these proteins (Figure 6G; Figure 2B, and Figure S15A). Additionally, luciferase complementation imaging (LCI) assays also exhibited these interactions, demonstrating significant luminescence increase when CsCBF1‐nLUC or CsCBF3‐nLUC was paired with CsCCR1‐cLUC (Figure 6H). These results cooperatively revealed a physical interaction between CsCCR1 and CsCBF1/CsCBF3, and these interactions were enhanced by low temperature (Figure S16A–D).

To further define the structural basis and specificity of the interaction, we performed CsCBF family sequence alignments and domain truncation assays. Sequence alignment showed that CsCBF proteins share a highly conserved AP2 DNA‐binding domain and two nuclear localization signals, but their N‐ and C‐terminal regions are highly divergent (Figure S17A). For truncation assays, we focused on key domains of CsCCR1 (ZF‐HD_dimer domain and homeo_ZF_HD domain) and CsCBF1/3 (AP2 domain and N/C‐terminal regions) (Figure S17B). Y2H and BiFC assays demonstrated that the C‐terminal homeo_ZF_HD domain of CsCCR1 interacted with the AP2 domain and C‐terminal of CsCBF1, and specifically interacted with the AP2 domain of CsCBF3 (Figure S17B,C).

To further examine the functional significance of these interactions, we performed promoter activation assays. The results showed that co‐expression of CsCCR1 with CsCBF1/3 (CsCCR1+CsCBF1+CsCBF3) significantly enhanced the LUC/REN signals of *pCsCHS1*, *pCsFLS1*, *pCsSCPL4*, *pCsSCPL5*, and *pCsELIP1* by 2.5‐fold compared to CsCCR1 alone, by 1.8‐fold compared to CsCCR1+CsCBF1, and 1.5‐fold compared to CsCCR1+CsCBF3 (Figure 6I). This synergistic activation indicates that CsCCR1 forms a transcriptional complex with CsCBF1 and CsCBF3 to enhance the expression of catechins biosynthetic genes in response to cold stress in tea plants.

### 2.7 CsLUX and CsKUA1 Directly Bind to the Promoter of *CsCCR1* and Activate Its Expression

**FIGURE 7:**
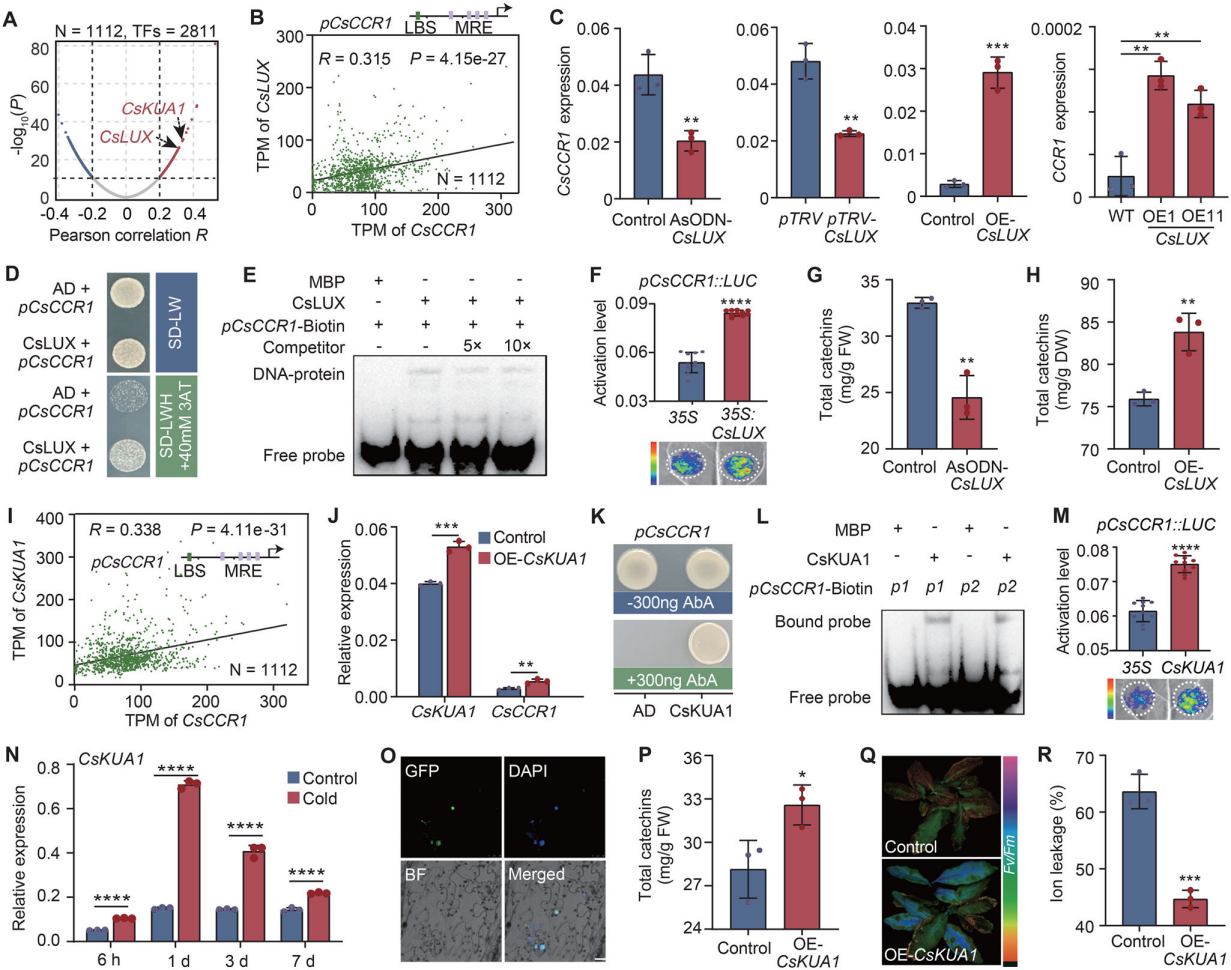
CsLUX and CsKUA1 directly upregulate the transcription of *CsCCR1*. (A) Expression correlation of *CsCCR1* with 2 811 transcription factors in 1 112 tea plant accessions. (B) TPM (transcripts per kilobase per million mapped reads) distribution of *CsCCR1* and *CsLUX* in 1112 tea accessions. (C) *CsCCR1* expression in *CsLUX*‐silenced and *CsLUX*‐overexpressing (OE) tea plants compared to controls, and *AtCCR1* expression in *CsLUX*‐OE *Arabidopsis* versus WT. (D) Y1H assay showing CsLUX binding to the *pCsCCR1*. L, Leucine; W, Tryptophan; H, Histidine. (E) EMSA confirms CsLUX binding to the LBS sites in *pCsCCR1*. (F) LUC phenotype and quantification. (G,H) Total catechins accumulation in *CsLUX*‐silenced and *CsLUX*‐overexpressing (OE) tea plants compared to controls. (I) TPM distribution of *CsCCR1* and *CsKUA1* in 1112 tea accessions. (J) Expression level of *CsKUA1* and *CsCCR1* in *CsKUA1*‐OE plants. (K–M) CsKUA1 binding to the promoter of *CsCCR1* and activates its transcription. (N) Expression level of *CsKUA1* under cold stress. (O) CsKUA1 localized in the nucleus. BF, bright field. Scale bars = 40 µm. (P–R) Cold tolerance and total catechins accumulation in *CsKUA1*‐OE plants. Data represent Mean ± SD, n = 3. Significance was determined by Student's *t*‐tests (^*^ *p* < 0.05, ^**^ *p* < 0.01, ^***^ *p* < 0.001, ^****^ *p* < 0.0001).

To understand the transcriptional regulation of CsCCR1, we first identified its potential upstream regulators by analyzing a large transcriptome dataset from 1 112 tea accessions. Co‐expression network analysis revealed that the expression of *CsCCR1* is highly correlated with two transcription factors, *CsLUX^2^* (*p* = 4.15e‐27) and CsKUA1 [59] (*p* = 4.11e‐31), among 2 811 transcriptional factors examined (Figure 7A,B,I). Sequence annotation indicated that *CsLUX* is a documented cold‐responsive MYB transcription factor [2], while *CsKUA1* represents a novel cold‐responsive MYB transcription factor, with both genes being significantly induced by cold stress (Figure S15B). These findings prompted us to investigate whether CsLUX and CsKUA1 directly regulate *CsCCR1*.

Our results showed that silencing *CsLUX* in tea leaves led to reduced *CsCCR1* expression, whereas overexpression of *CsLUX* in both tea seedlings and *Arabidopsis* resulted in elevated transcript levels of *CsCCR1* and its ortholog *AtCCR1*, respectively (Figure 7C). Similar to the expression pattern of *CsLUX*, *CsCCR1* expression and catechins accumulation exhibited diurnal rhythmicity (Figure S18). Using a combination of in vivo (Y1H, LUC) and in vitro (EMSA) assays, we demonstrated that CsLUX could directly bind to the promoter of *CsCCR1* and activate its expression (Figure 7D–F). Furthermore, overexpression of *CsLUX* upregulated the expression of five key genes in catechins biosynthesis (*CsCHS1*, *CsFLS1*, *CsSCPL4*, *CsSCPL5*, and *CsELIP1*), while silencing *CsLUX* downregulated their expression, leading a differentially catechins accumulation (Figure 7G,H and Figure S19A–D). These results collectively demonstrated that CsLUX directly activates the expression of *CsCCR1* to regulate catechins biosynthesis in tea plants.

In addition to the binding sites of LUX (LBS, GATA/TCG), we also found four MYB‐related *cis*‐elements in the promoter of *CsCCR1* (Figure 7I). A strong positive correlation was also observed between the expression of *CsCCR1* and *CsKUA1* (MYB transcription factor) across 1 112 tea accessions (*r* = 0.338, *p* = 4.11e‐31). Consistently, overexpression of *CsKUA1* in tea seedlings led to an elevated *CsCCR1* transcript level, indicating their potential regulatory relationship (Figure 7J). Subsequent experiments using Y1H, EMSA, and LUC assays demonstrate that CsKUA1 directly binds to the promoter of *CsCCR1* and activates its expression (Figure 7K–M).

To further examine whether CsKUA1 regulates catechins biosynthesis and cold tolerance in tea plants, we first investigate its subcellular localization and expression patterns in representative tissues. The results found that CsKUA1 is a nuclear‐localized transcription factor, whose expression is strongly induced by cold stress (4.71‐fold) and is highly expressed in young buds and leaves (Figure 7N,O and Figure S20A). Functional studies further showed that overexpression of *CsKUA1* not only activated the expression of key catechins biosynthetic genes (*CsCHS1*, *CsFLS1*, *CsSCPL4*, *CsSCPL5*, and *CsELIP1*), but also enhanced cold tolerance of tea seedlings (Figure 7P–R and Figure S20B,C). Taken together, these results suggested that both CsLUX and CsKUA1 positively regulated cold tolerance and catechins accumulation by directly activating *CsCCR1* expression.

### 2.8 CsCCR1 Mediates Catechins Biosynthesis to Improve Cold Resistance in Tea Seedlings by Modulating ROS Homeostasis

To further elucidate how *CsCCR1*‐mediated catechins biosynthesis enhances cold resistance in tea plants, we first performed transcriptome analysis of *CsCCR1*‐overexpressing plants. This identified 787 DEGs, predominantly enriched in the hydrogen peroxide metabolic process (GO: 0042743), reactive oxygen species (ROS) metabolic process (GO: 0072593), response to oxidative stress (GO: 0006979), and phenol‐containing compound metabolic process (GO: 0018958) (Figure S21A). Besides, our results also showed that the expression levels of ROS scavenging genes were significantly upregulated in *CsCCR1*‐overexpressing plants (Figure S21B). These results indicate that *CsCCR1* may mediate catechins biosynthesis to modulate ROS levels in response to cold stress in tea plants.

**FIGURE 8:**
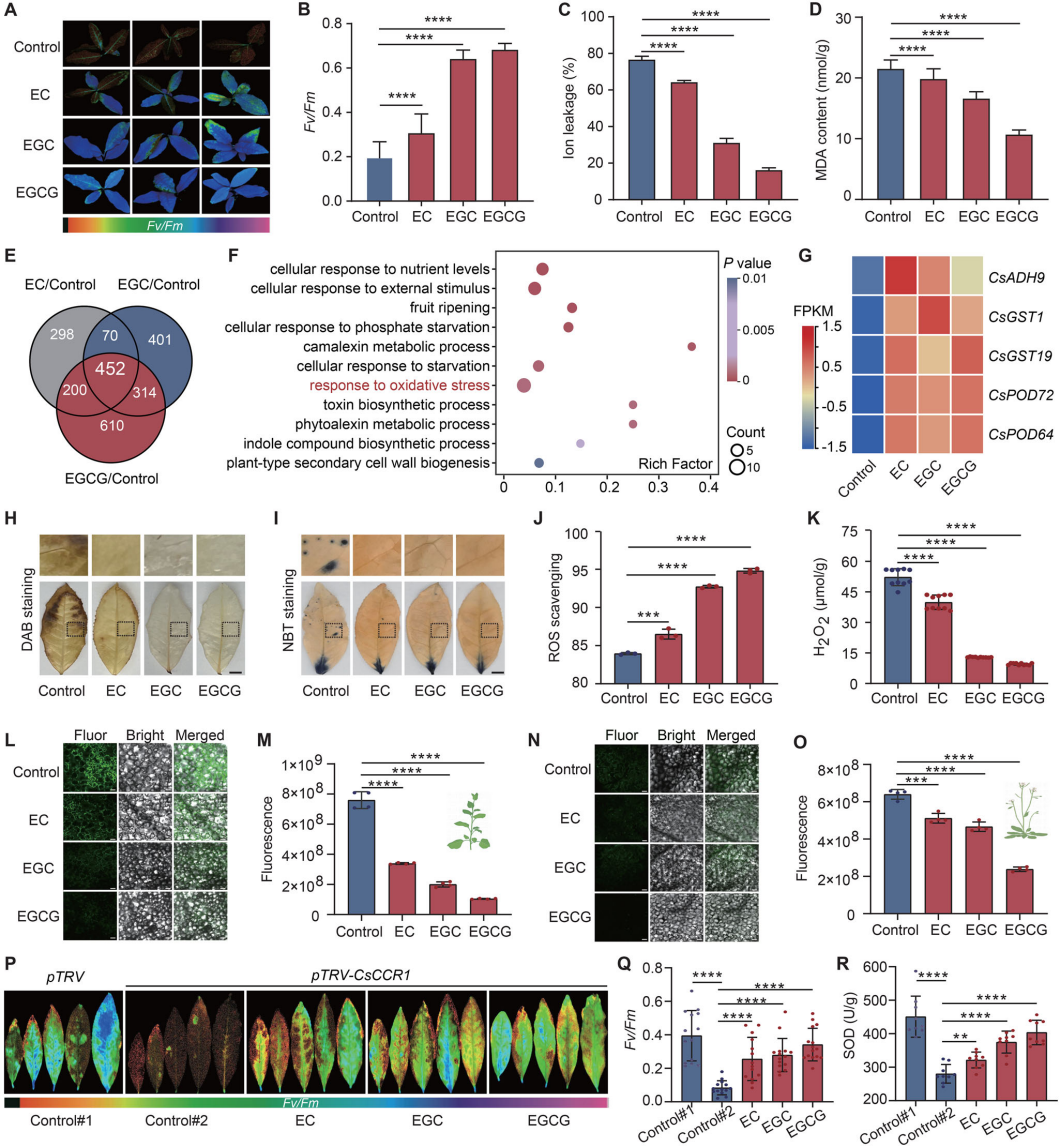
EGCG, EGC, and EC applications enhance cold tolerance via the cellular ROS level. (A–D) Phenotypic and physiological responses (*Fv/Fm*, ion leakage, and MDA levels) of tea seedlings treated with 1 mm EC, EGC, and EGCG under freezing stress. (E) Venn plot of 452 public DEGs for EC/EGC/EGCG versus control. (F) GO enrichment analysis of 452 DEGs of EC/EGC/EGCG versus control. (G) Normalized FPKM of ROS scavenging genes. (H,I) DAB staining for H_2_O_2_ and NBT staining for O^2−^. Scale bars = 0.5 cm. (J,K) ROS scavenging ability and the contents of H_2_O_2_ in treated samples. (L,M) Imaging and quantification of the fluorescence intensity detected with ROS probes H_2_DCFDA in tobacco treated with 1 mM EC, EGC, and EGCG under cold stress. Scale bars = 40 µm. (N,O) Imaging and quantification of the fluorescence intensity detected with ROS probes H_2_DCFDA in *Arabidopsis* treated with 1 mm EC, EGC, and EGCG under cold stress. Scale bars = 40 µm. (P–R) Exogenous EC, EGC, and EGCG application rescues the cold damage in *CsCCR1*‐silenced tea seedlings. The color bar represents damage severity via *Fv/Fm* value, with reduced damage from left to right. All data in the barplot were denoted as Mean ± SD, n = 3. Statistical significance was determined by Student's *t*‐tests (^*^ *p* < 0.05, ^**^ *p* < 0.01, ^***^ *p* < 0.001, ^****^ *p* < 0.0001).

To further examine the role of catechins in cold tolerance, we also performed foliar applications of EGCG, EGC, and EC on tea seedlings. The results suggested that tea seedlings treated with 1 mm EC, EGC, and EGCG exhibited enhanced freezing tolerance, showing higher *Fv/Fm* but lower ion leakage and MDA than the control group (Figure 8A–D and Figure S22). Furthermore, transcriptomic profiling identified 452 consensus DEGs across tea leaves treated EC/EGC/EGCG compared to the control (Figure 8E). These DEGs were predominantly enriched in oxidative stress pathways (8.19% DEGs in GO: 0006979, *p* = 1.55e‐5), further suggesting that tea catechins may enhance cold tolerance of tea plants by modulating ROS accumulation (Figure 8F,G).

To validate this, we performed 3,3′‐diaminobenzidine (DAB) and nitroblue tetrazolium (NBT) staining on tea leaves treated with EC, EGC, or EGCG under cold stress. The results showed shallow staining intensity in catechin‐treated leaves compared to the blank control, indicating reduced ROS accumulation (Figure 8H,I). Consistently, these leaves also exhibited enhanced ROS scavenging ability and lower H_2_O_2_ levels than the blank control (Figure 8J,K). In addition, the fluorescence intensity in leaves of *N. benthamiana* and *Arabidopsis* stained with the ROS probe H_2_DCFDA was observed and quantified, and both results suggested that plants with EC, EGC, or EGCG applications had significantly lower levels of ROS signal than the control under cold stress, especially EGCG (Figure 8L–O). Interestingly, exogenous application of EC, EGC, and especially EGCG significantly rescued the cold‐sensitive phenotype of *CsCCR1*‐silenced tea seedlings, with enhanced *Fv/Fm* and SOD activity (Figure 8P–R). Together, these findings suggested that *CsCCR1* orchestrates EGCG, EGC, and EC accumulations to alleviate cold‐induced oxidative damage and thereby enhancing cold tolerance of tea leaves.

To further explore the mechanism by which catechins reduce ROS levels, we examined the enzymatic (SOD, POD, CAT, APX) and non‐enzymatic (GSH and AsA) antioxidants in tea plants with silenced or overexpressed catechins‐biosynthetic genes, as well as in catechin‐treated plants. The results showed that compared with controls, the activities of SOD, POD, CAT, and APX, as well as the contents of GSH and AsA, were significantly reduced in *CsCHS1/FLS1/SCPL4/5/ELIP1*‐silenced tea plants (Figure S23A). Conversely, all of these ROS‐related indicators were markedly increased in corresponding gene‐overexpressing tea plants (Figure S23B). Moreover, exogenous applications of EC, EGC, and particularly EGCG substantially elevated the activities of these antioxidant enzymes and the accumulation of GSH and AsA (Figure S23C). These results demonstrated that beyond acting as direct antioxidants, catechins might also function as signaling molecules that coordinately activate the endogenous ROS‐scavenging machinery, including both enzymatic (SOD, POD, CAT, APX) and non‐enzymatic (GSH, AsA) antioxidant pathways.

### 2.9 Natural Variation of *CsCCR1* Enhances Catechins Accumulation and Cold Tolerance in Tea Plants

**FIGURE 9:**
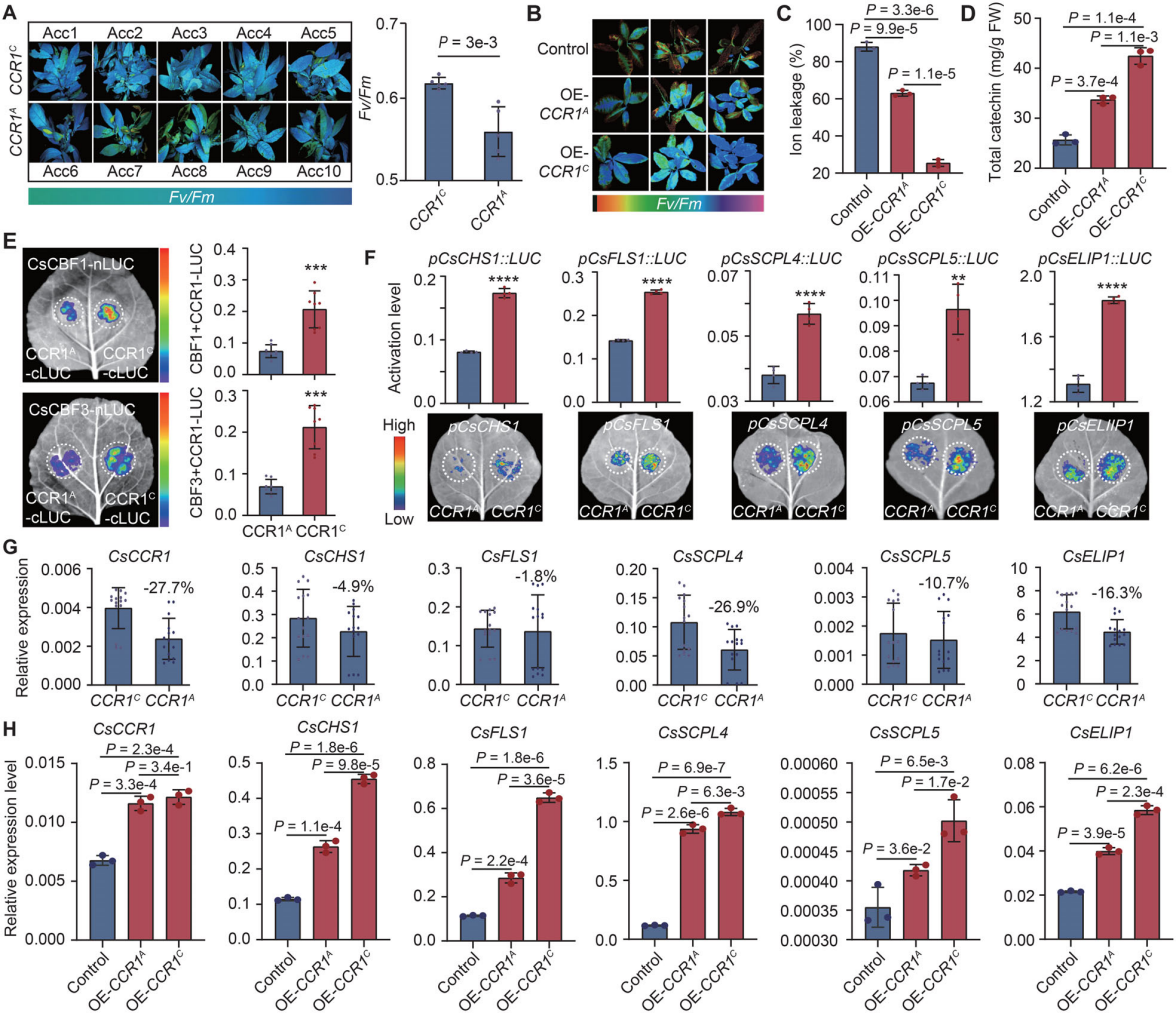
Natural variation in *CsCCR1* contributes to cold tolerance and catechins biosynthesis. (A) Chlorophyll fluorescence imaging and photosynthetic efficiency of five random tea accessions in each haplotype of *CsCCR1* under cold stress. (B) Chlorophyll fluorescence estimation of tea seedlings treated with −5°C for 30 min. The color bar represents damage severity, with severe damage from left to right. (C) Ion leakage of OE‐*CsCCR1^A^*, OE‐ *CsCCR1^C^* and control lines under cold stress. (D) Total catechins accumulation in OE‐*CsCCR1^A^*, OE‐ *CsCCR1^C^* and control lines. FW: fresh weight. (E) LCI showing higher binding affinity of CsCBF1/3 and CsCCR1^C^ than CsCCR1^A^. (F) Dual‐LUC showing higher activation ability CsCCR1^C^ on downstream target genes than CsCCR1^A^. (G) Expression levels of *CsCCR1* and its target genes in OE‐*CsCCR1^A^*, OE‐ *CsCCR1^C^* and control lines. (H) Expression levels of *CsCCR1* and its target genes in two *CsCCR1* genotypes. Alll data in the barplot were denoted as Mean ± SD, n = 3. Statistical significance was determined by Student's *t*‐tests (^**^ *p* < 0.01, ^***^ *p* < 0.001, ^****^ *p* < 0.0001).

Integrated genomic, physiological, and metabolomic analyses revealed that a missense mutation (A‐to‐C) in coding region of *CsCCR1* is associated with superior cold tolerance and elevated accumulation of major catechins (EGCG, EGC, EC) in natural tea populations (Figure 1E–O and Figure S7). To further functionally characterize this natural variation, we examined the cold tolerance of *CsCCR1^A^* and *CsCCR1^C^* in ten randomly selected accessions. We showed that the tea accessions carrying *CsCCR1^C^* maintained higher photosynthetic performance under cold stress than those carrying *CsCCR1^A^* (Figure 9A). We further generated transgenic tea seedlings overexpressing either the *CsCCR1^A^* or *CsCCR1^C^* genotypes. Results also showed that *CsCCR1^C^* overexpression lines displayed enhanced cold tolerance relative to *CsCCR1^A^* lines under cold stress, as evidenced by higher *Fv*/*Fm* values and lower ion leakage (Figure 9B,C and Figure S24A). The results consistently showed that *CsCCR1^C^* overexpressing lines accumulated more total catechins as well as individual catechins monomers such as EGCG, EGC, EC, and ECG than *CsCCR1^A^* lines (Figure 9D and Figure S24B). Together, these results collectively demonstrate that the *CsCCR1^C^* enhances both catechins accumulation and cold tolerance in tea plants.

To dissect the underlying mechanism, we first predicted the structural changes of CsCCR1^A^ and CsCCR1^C^ proteins. The results showed that the A to C variation leads to a Q to P amino acid substitution located between two conserved domains of CsCCR1. This substitution altered the local backbone conformation and interaction interface of CsCCR1, resulting in obvious overall structural changes with an RMSD (root mean square deviation) value of 29.208, which was mainly reflected in reduced distances among adjacent amino acid residues (Figure S25A,B). Molecular docking further indicated that the CsCCR1^A^ to CsCCR1^C^ missense variation increased the number of interaction bonds between CsCCR1 and CsCBF1/3 (Figure S25C). We subsequently conducted BiFC assays to semi‐quantitatively verify the differences in interaction activities. Results demonstrated stronger fluorescence signals or activities for CsCCR1^C^‐CsCBF1/3 interactions than CsCCR1^A^ (Figure S25D,E and Figure 9E). SPR assays also showed that CsCCR1^C^ had a lower K_D_ value for CsCBF1 than CsCCR1^A^, indicating a stronger binding affinity between CsCBF1 and CsCCR1^C^ (Figure S25F). This enhanced binding capability promoted more potent transcriptional activation of downstream target genes (*CsCHS1*, *CsFLS1*, *CsSCPL4*, *CsSCPL5*, and *CsELIP1*) by the *CsCCR1^C^* genotype compared to *CsCCR1^A^* genotype (Figure 9F), thereby explaining the genotype‑dependent expression pattern in natural populations. The overexpression lines of the *CsCCR1^C^* genotype also exhibited significantly higher expression of those catechins biosynthetic genes than *CsCCR1^A^* lines (Figure 9G). These results demonstrated that the natural mutation in *CsCCR1* functions as a key polymorphism that coordinately enhances catechins biosynthesis and cold tolerance in tea plants through a CBF‐mediated regulatory pathway.

## 3 Conclusion

**FIGURE 10:**
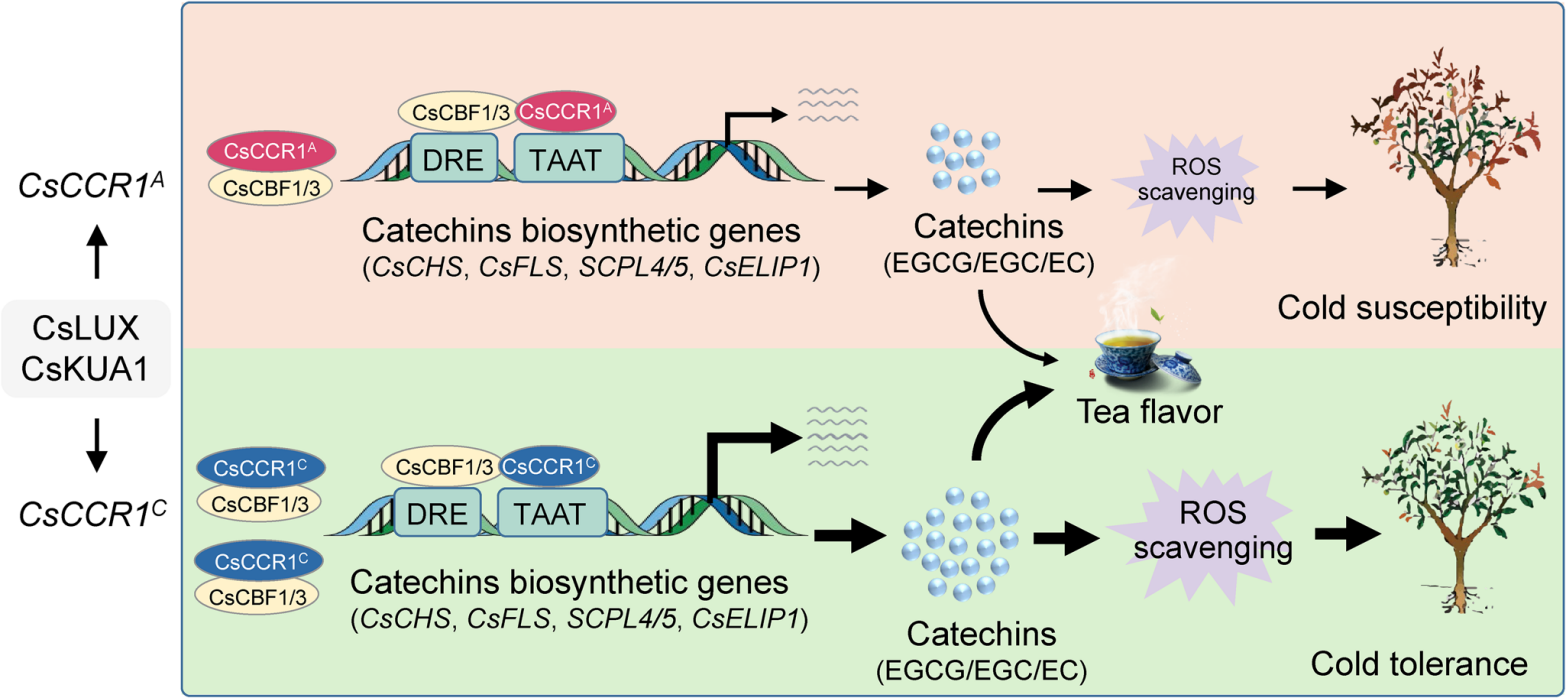
A *CsCCR1*‐regulated module on cold tolerance and catechins accumulation in tea plants.

The thermophilic nature of tea plants, coupled with increasing consumer demand for health‐oriented tea products, underscores the urgent need to elucidate mechanisms underlying cold tolerance and tea quality formation. In this study, we established the panel providing CTI and twelve tea‐quality‐related metabolites in 108 tea accessions with highly geographic and genetic diversity. We integrated genome‐wide associated studies with functional characterization to identify *CsCCR1* as a key transcription factor that coordinately enhances cold tolerance and promotes the biosynthesis of major tea catechins (EGCG, EGC, and EC). We further deciphered a synergistic regulatory module wherein CsCCR1 interacts with CsCBF1/3 to active catechin biosynthetic genes (*CsCHS1*, *CsFLS1*, *CsSCPL4*, *CsSCPL5*, and *CsELIP1*) under cold stress (Figure 10). This regulatory cascade is initiated by the cold‐responsive transcription factors CsLUX and CsKUA1, which directly activate *CsCCR1* expression. Crucially, we demonstrated that the accumulated catechins, particularly EGCG, significantly enhance cold tolerance of tea plants by strengthening the ROS scavenging capacity, thereby maintaining cellular homeostasis under cold stress. Importantly, the natural variation of *CsCCR1* significantly altered the catechins accumulation and cold tolerance of tea accessions, with high catechins accumulation and cold tolerance in CC genotype. Our findings provide novel insights into the transcriptional coordination of cold tolerance and secondary metabolism in perennial crops, offering potential strategies for breeding climate‐resilient tea varieties with improved quality in the future.

## 4 Discussion

### 4.1 Cold Tolerance and Secondary Metabolite Diversification in Tea Population

To dissect the genetic basis of cold tolerance, we established a robust phenotyping framework by systematically evaluating 108 diverse tea accessions collected from 12 major tea‐producing regions across China. This collection captures a broad geographic and genetic diversity, providing a valuable resource for genetic studies. Unlike annual grain crops and some horticultural species whose cold tolerance is typically assessed by survival rate under low temperatures, the tea plant is a perennial leaf‐harvested woody species, which requires a more nuanced approach [26, 27, 28, 29, 30]. Established methodology in tea plants mainly involves assessing leaf functionality and physiological integrity, typically through evaluation of leaf damage severity and physiological changes under controlled conditions [2, 4, 8]. In line with this paradigm, we here integrated field‐based overwintering phenotyping of leaf damage with laboratory quantification of four key physiological indices (REC, MDA, Pro content, and SOD activity) for tea plants treated under indoor controlled conditions. These multi‐dimensional data were incorporated into a weighted composite cold tolerance index (CTI), where REC emerged as the most influential parameter (explaining 34.14% of variance), aligning with its established role as a sensitive cellular stress biomarker [31, 32]. This integrated framework not only confirms the feasibility of our evaluation method but also provides a more holistic assessment than single‐indicator assays.

Besides, metabolic profiling also quantified 12 key secondary metabolites linked to tea quality, revealing substantial diversity in their accumulation across the population. Notably, we observed particularly high variation in key metabolite accumulation such as theobromine, theanine, and EGCG, which are crucial metabolites associated with both tea quality and cold stress response. It should be mentioned that, while most metabolites were accurately detected, trace compounds like C, GCG, and THB may still require refined UPLC methods for precise quantification. Nevertheless, our systematic traits analysis provides a foundation for understanding the genetic covariation of cold resilience and quality traits in tea plants. The collected tea accessions also exhibited extensive phenotypic and genetic diversity, serving as a valuable resource for GWAS in tea plants.

### 4.2 Dual Regulation of Cold Tolerance and Catechins Biosynthesis by *CsCCR1*

GWAS has been reported to be an effective approach to identify novel genes associated with some agronomic traits, significantly advancing our understanding of their genetic contributions to these important traits [33, 34, 35, 36, 37, 38, 39]. Using 13.38 million SNPs, GWAS pinpointed *CsCCR1* as a candidate regulator for both cold tolerance and catechins biosynthesis, especially EGCG, EGC, and EC, in tea plants. Functional studies confirmed that overexpression of *CsCCR1* improves cold resistance and elevates catechins levels, whereas its silence leads to reduced catechins accumulation and increased freezing sensitivity. While the conserved motif in CCR1 (homeobox) is known to regulate plant development and drought response [11, 40], our study reveals a previously undocumented role for *CsCCR1* in concurrently regulating cold stress response and catechins biosynthesis in tea plants. Besides, ROS are central to stress signaling but can trigger cytotoxicity at high levels [41, 42, 43, 44]. Here, we show that exogenous application of EC, EGC, and EGCG significantly bolstered cold tolerance of tea plants by alleviating ROS‐induced damage, with ROS‐scavenging capacity correlating with the degree of catechin hydroxylation (EGCG > EGC > EC). While EGCG has previously been linked to freezing stress response in tea plants [14], our results further demonstrated that EGC and EC, as critical ROS scavengers, also enhanced cold tolerance of tea seedlings—a finding with potential applications in tea breeding and functional food development. Moreover, given existing evidence of crosstalk between catechins and phytohormone (JA/ABA/SA) pathways [45, 46, 47, 48, 49], further studies should investigate whether *CsCCR1* serves as an integrative node coordinating catechins biosynthesis and hormone signaling to balance growth and stress response in tea plants.

### 4.3 Synergistic Transcriptional Activation of Catechins‐related Genes by CsCCR1 and CsCBF1/3

Our study reveals a novel transcriptional regulatory module in which the homeodomain protein CsCCR1 and DREB transcription factor CsCBF1/3 work together to coordinately activate catechin biosynthesis and cold adaptation in tea plants. While CCR and CBF family members have independently been implicated in tea bud dormancy and cold response previously [12], we provide the first evidence of their functional synergy in regulating specialized metabolism under cold stress in tea plants. Mechanistically, CsCCR1 specifically binds to the TAAT motifs in the promoters of key catechins‐biosynthetic genes (*CsCHS1*, *CsFLS1*, *CsSCPL4*, *CsSCPL5*, and *CsELIP1*), while CsCBF1/3 bind to its adjacent DRE elements in these promoters. These results revealed that the binding elements of CCR (TAAT) and CBF (A/GCCGAC) are identical in both *Arabidopsis* and tea plants [8, 43, 50, 51]. A previous report suggested CBF2 indirectly regulated the expression of *BcF3H/BcFLS1* in flavonla biosythesis, which depends on the transcriptional activation of CBF2 on MYB111 in cabbage [52]. Ulike *BcCBF2‐BcMYB111‐BcF3H/BcFLS1* pathway in cabbage, we identified a direct enhancement of CsCBF1/3 in flavonol biosynthesis. Beyond the simple regulation of *FveDREB1B* on *FveCHS* in strawberry [24], our regulatory module of *CsCBF1/CBF3‐CsCHS1/FLS1/SCPL4/SCPL5/ELIP1* provides a broader understanding of how CBFs regulate anthocyanin biosynthesis.

Notably, the discovery of CsCCR1‐CsCBF1/3 interaction represents a significant advance in understanding transcriptional regulation in plant specialized metabolism, exhibiting both similarities and distinct differences compared to canonical regulatory modules such as MYB‐bHLH‐WD40 complexes in anthocyanin biosynthesis [53, 54, 55, 56]. In parallel with the activation of MYB‐bHLH‐WD40 complex on expression of DREB1 in rice [57], our results also showed CsLUX and CsKUA1, being MYB transaction factor, activate *CsCCR1* expression in tea plants. These results suggest that the CBF‐dependent and CBF‐independent pathway together leads to catechins biosynthesis, which is conserved across species. Together, the *CsLUX/KUA1‐CsCCR1/CBF1/CBF3‐CsCHS1/FLS1/SCPL4/SCPL5/ELIP1* regulatory hierarchy offers a novel and comprehensive molecular module in catechins biosynthesis in tea plants.

### 4.4 Functional Convergence of Catechins Biosynthesis and Cold Tolerance in Tea Plants

This study identifies *CsCHS1*, *CsFLS1*, *CsSCPL4*, *CsSCPL5*, and *CsELIP1* as core components coordinately linking catechins biosynthesis and cold tolerance in tea plants. Among these, *CsCHS1*, *CsFLS1*, *CsSCPL4*, and *CsSCPL5* were previously known to participate in catechins biosynthesis [55]. Their predominant expression, along with that of *CsCCR1*, in apical buds and young leaves coincides with the established catechins accumulation profiles [22], suggesting the spatially coordinated regulation mediated by *CsCCR1*. Notably, key catechins pathway genes, such as *CHS1*, *F3’H*, *LAR*, have been implicated in cold tolerance via anthocyanins metabolism [53, 58], underscoring the crosstalk between catechins biosynthesis and cold response in plants. Beyond these transcriptional regulations, catechins biosynthetic genes are also regulated at the post‐transcriptional levels. Fine‐tune mechanisms, such as microRNA‐mediated regulation of CHS, long non‐coding RNA interactions with MYB, and ubiquitin modification of MYB transcription factors, may represent efficient strategies evolved in plants to modulate metabolite biosynthesis and stress response [53, 59, 60].

Another advance of this study is the discovery of *CsELIP1* as a novel cold‐induced regulator of EGCG biosynthesis in tea plants. Its expression is dramatically induced (28‐fold) under cold stress and is regulated by CsCCR1, suggesting a potentially unique adaptation mechanism of tea plants that coordinates stress response with specialized metabolite production. Unlike the sustained induced expression pattern observed for *CsELIP1* under cold stress, its homolog in *Arabidopsis*, known as early light‐induced protein 1 (ELIP1), plays significant roles in regulating chlorophyll content, phytochrome signaling, and light‐dependent pigment metabolism [61]. Interestingly, our data also found that the key biosynthetic enzymes of catechins (CsCHS1 and CsFLS1) reside in the cytoplasm and nucleus, while CsELIP1 is chloroplast‐localized. It is intriguing how chloroplast‐localized CsELIP1 could influence nuclear EGCG accumulation. A mechanistic explanation supported by the literature is that phenolic and flavonoid intermediate metabolites can dynamically shuttle between chloroplasts and the nucleus [62]. Chloroplasts act as a primary source of upstream shikimate pathway‐derived precursors, which can be transported into the nucleus to serve as substrates for downstream EGCG biosynthesis catalyzed. As a chloroplast‐localized regulator, CsELIP1 may modulate the biosynthesis or export of these upstream precursors in chloroplasts, thereby indirectly altering substrate availability for nuclear catechin metabolism and ultimately affecting nuclear EGCG content. This compartmentalized metabolic pattern is also consistent with the findings that catechin compounds naturally accumulate in both chloroplasts and nuclei in tea plant leaves [63], and emerging evidence has further demonstrated the nucleocytoplasmic trafficking of core phenylpropanoid biosynthetic enzymes to coordinate subcellular flavonoid production [64].

### 4.5 CsLUX and CsKUA1 Modulate *CsCCR1* to Respond Cold Stress and Promote Catechins Biosynthesis in Tea Plants

Our previous study has shown that *CsLUX*, a core component of the circadian clock, could enhance cold tolerance in tea plants [2]. Similarly, *CsKUA1*, known to influence ROS homeostasis and chloroplast biogenesis, has been reported to improve cold tolerance in rice [65, 66, 67]. However, the molecular mechanism underlying their roles in cold tolerance remains unclear. In this study, large‐scale transcriptome analysis across 1 112 tea accessions provided strong evidence for a functional association among *CsCCR1*, *CsLUX*, and *CsKUA1*. We demonstrated that *CsCCR1* acts as a direct target of both CsLUX and CsKUA1, and that these transcription factors regulate the expression of key catechin biosynthetic genes (*CsCHS1*, *CsFLS1*, *CsSCPL4*, *CsSCPL5*, and *CsELIP1*) through *CsCCR1*‐mediated transcription (Supplemental Figure 15). These findings offer novel mechanistic insights into how *CsLUX* and *CsKUA1* regulate cold stress and catechins biosynthesis in perennial crops. Interestingly, both *CsLUX* and *CsKUA1* homologs in other species are known to participate in light‐mediated hypocotyl elongation [68, 69, 70, 71]. This raises an intriguing possibility that *CsCCR1* may serve as a convergence point integrating light singling with catechins accumulation and cold tolerance in tea plants—a hypothesis that warrants further investigation.

Given that *CsLUX* is a known component of the circadian clock, we further analyzed the expression of *CsCCR1* and *CsLUX* every 4 h for two consecutive days. As expected, *CsCCR1* expression exhibited diurnal rhythmicity under a normal growth environment, peaking at dusk, which has a similar expression pattern with *CsLUX* (R = 0.375, *p* = 1.86e‐2; Figure S18) [2]. Similar to *CsLUX* expression, *CsCCR1* expression was elevated under cold stress. Unlike the maintained expression amplitude of *LUX* in *Arabidopsis*, cold stress disrupted the diurnal rhythmicity cycles of *CsLUX* and *CsCCR1* expression [72] in tea plants. Correspondingly, we also detected the total catechins, EGCG, EGC, and EC accumulations every 4 h for two consecutive days. The highest accumulations of these catechins were detected around ZT8‐12 h and ZT32‐36 h, while the lowest accumulations of these catechins were detected around ZT24 h. These rhythmic accumulations of catechins mirrored to *CsLUX* and *CsCCR1* expression, which expands upon the accumulation pattern of catechins in a previous report [73]. These indicate the differences in cold tolerance at different times of the day, which need further investigation.

### 4.6 A Missense Mutation in Coding Region of *CsCCR1* Pleiotropically Regulates Catechin Biosynthesis and Cold Tolerance in Tea Plants

This study also identifies a functionally significant missense mutation (A356C) in the coding sequencing of *CsCCR1* that coordinately enhances both catechins accumulation (EGCG, EGC, and EC) and cold tolerance in *Camellia sinensis*. Population studies revealed that the *CsCCR1* ^C^ genotype is consistently associated with superior cold tolerance and elevated levels of major catechins (EGCG, EGC, EC) compared to *CsCCR1* ^A^ genotypes (Figure S7A,B). Transgenic validation further confirmed the functional significance of this natural variation on cold tolerance and catechins biosynthesis in tea plants. Tea plants overexpressing the *CsCCR1* ^C^ allele exhibited higher accumulation of EGCG, EGC, EC, and ECG than those overexpressing the *CsCCR*1^A^ allele (Figure 9). The *CsCCR1* ^C^ also exhibited mild freezing symptoms compared to severe injury in *CsCCR1* ^A^. From a breeding perspective, the dual functionality of the *CsCCR1* ^C^ offers a valuable genetic resource for precision improvement in tea plants. Marker‐assisted introgression of the *CsCCR1* ^C^ genotypes could accelerate the development of premium tea cultivars with enhanced cold hardiness and catechins content — traits of particular importance for high‐altitude cultivation. Nevertheless, how this natural variation in the regulatory networks through which *CsCCR1* modulates cold tolerance and catechins biosynthesis needs further investigation.

## 5 Methods

### 5.1 Plant Materials and Growth Conditions

A diverse panel of 108 tea accessions used for genome‐wide association studies was extensively collected from 12 major tea‐producing provinces across China to maximize genetic and geographical representation. All accessions were consistently cultivated and maintained at the Anhui Academy of Agricultural Sciences Germplasm Repository. For genome sequencing and metabolite detection, healthy young leaves were sampled from each accession and then immediately frozen in liquid nitrogen and stored at −80°C. For cold tolerance assessment, uniform cuttings from all accessions were first rooted and then transplanted into seedling trays. These trays were subsequently placed in a temperature‐controlled chamber for the standardized cold treatment prior to sampling to assess cold tolerance. To mitigate microenvironmental variation, the field was uniformly tilled and homogenized prior to planting, and experimental plots were placed inside guard rows to reduce spatial variation and biotic interference. Daily temperature data for both years were obtained from website of “www.tianqi.com”. A summary of daily temperature ranges and low‐temperature event durations for each year was provided in Data S8.

For gene cloning, exogenous feeding assays, gene silencing, and overexpression, the tea cultivar “shuchazao” (*C. sinensis* var. *sinensis* “Shuchazao”) was used. For physiological analyses, relative electrical conductivity (REC), malondialdehyde (MDA), proline (Pro), and superoxide dismutase (SOD) were quantified following freezing treatment. For heterologous functional studies, *Arabidopsis thaliana* (ecotype Columbia‐0) was used as the wild‐type control, and transgenic lines were generated using the floral dip method [74]. *Nicotiana benthamiana* was served as the host for subcellular localization, dual‐luciferase, and luciferase complementation imaging assays. Seeds of both species were surface‐sterilized, vernalized for 3 days at 4°C, and germinated on 1/2 Murashige and Skoog (MS) medium containing 0.8% agar plate or nutrient soil mined with 1:1 vermiculite in climate‐controlled chambers.

### 5.2 Evaluation of Cold Tolerance in Tea Plants

Cold tolerance of 108 tea accessions was assessed using a combination of field‐based overwintering observations of leaf damage and controlled laboratory physiological assays, with cross‐validation between these methods ensuring comprehensive and reliable evaluation. Field‐based overwintering assessment was conducted following the standardized protocol outlined in *Evaluation Standards for Elite and Rare Germplasm Resources—Tea Plant (Camellia sinensis (L.) O. Kuntze)* (NY/T 2031–2011) over two consecutive years. Briefly, cold damage of tea leaves was investigated in 10 plants per accession after natural cold events. A leaf was considered cold‐damaged if more than one‐third of its area exhibited reddish or bluish withering. Cold injury was graded on a 0–4 scale based on the percentage of damaged leaf area: level 0 (≤ 5%), level 1 (6%–15%), level 2 (16%–25%), level 3 (26%–50%), and level 4 (> 50%).

To further complement field observations and enable standardized physiological assessment, uniform cuttings from all 108 accessions were rooted and transplanted into seedling trays, which were then subjected to controlled cold treatment at −5°C for 2 h in an artificial climate chamber. Following treatment, four key physiological parameters associated with cold tolerance were measured. The REC was measured using a previously described method after cold stress [2]. The levels of Pro, MDA, H_2_O_2_, and SOD activity were measured using Proline Content Detection Kit (BC0250), Malondialdehyde Content Detection Kit (BC0020), Hydrogen Peroxide Content Detection Kit (BC3590), and Superoxide Dismutase Activity Assay Kit (BC0170), following the manufacturer's protocol. A Composite Cold Tolerance Index (CTI) was developed by integrating these four physiological parameters according to previous reports [26, 27, 28]. Weighting coefficients were determined using a combined approach of the Criteria Importance Through Intercriteria Correlation method and the Analytic Hierarchy Process. The CTI calculation involved normalization of each parameter followed by weighting integration. Best linear unbiased estimates of CTI across two consecutive years were used to represent the comprehensive cold tolerance of each accession. Crucially, the CTI values showed strong correlation with field‐based cold injury indices, demonstrating the reliability of this multi‐dimensional assessment in cold tolerance of tea plants.

### 5.3 Determination of Catechin Contents

Fresh tea samples for catechins extraction and quantification were immediately frozen in liquid nitrogen and ground to a fine powder, with three biological replicates per sample. For each replicate, either 0.10 g of fresh tissue or 30 mg of freeze‐dried powder was homogenized with 80% (v/v) methanol. The homogenate was subjected to ultrasonic‐assisted extraction for 30 min, followed by centrifugation at 10000 g for 10 min at 4°C. The supernatant was filtered using a 0.22 µm microfilter prior to HPLC analysis.

Catechins were quantified using a Waters ArcHPLC system equipped with a Shim‐pack GIS C18 reverse‐phase column. The mobile phase consisted of 0.1% Acetic acid in water and acetonitrile, with a flow rate of 1.0 mL/min. Detection was performed at 275 nm using a photodiode array detector. Individual catechin compounds (EGCG, EGC, EC, and ECG) were identified by comparing their retention times with authentic standards and quantified based on standard calibration curves. For cold‐induced upregulation ratios of catechins, the calculation formula: upregulation ratio (%) = (C_cold_‐C_control_)/C_control_×100%. Where C_control_ is the catechin content under normal temperature, and C_cold_ is the catechin content under cold treatment. For total catechins/EGCG/EGC/EC, C_cold_ is 112.17/47.71/38.89/10.87 mg.g^−1^, and C_control_ is 100.32/43.04/33.95/9.43 mg.g^−1^, thus, the enhanced ratio is 11.81/10.84/14.55/15.25%, respectively.

### 5.4 Sample Sequencing, Data Processing and SNP Calling

Genomic DNA was extracted from all 108 tea plant accessions and sequenced using the DNBSEQ sequencing platform, followed by quality control to remove adaptor‐contaminated and low‐quality reads. Clean reads were aligned to the reference genome (“Shuchazao”) using BWA‐MEM (v0.7.17) [75], with subsequent processing including reads sorting, mapping quality filtering (MAPQ ≥ 20), and reads duplicating using SAMtools (v1.12) [76]. Single nucleotide polymorphisms (SNPs) calling was conducted with GATK (v4.2.6.1) [77]. SNPs with only biallelic alleles, heterozygous rate < 0.25, missing rate < 0.1, and minor‐allele frequency > 0.1 were retained for population genetic analysis.

The SNP density was displayed using “CMplot” package implemented in R. The maximum‐likelihood (ML) phylogenetic tree was constructed using IQ‐TREE (v2.1.2) under the optimal substitution model TvM+e+ASC+R2 (1000 bootstraps), which was visualized using Figtree [78, 79]. Principal component analysis was conducted using EIGENSOFT (v7.2.1) based all SNPs [80]. The number of ancestral populations (*K*) of population structure was inferred using a cross‐validation approach by ADMIXTURE (v1.3.0) [81]. The linkage disequilibrium (LD) analysis was performed using LDBlockShow (v1.36) [82].

### 5.5 Genome‐wide Association (GWAS) Study

The genome‐wide association study was conducted using the filtered panel of 13 383 259 high‐quality SNPs (retaining only biallelic SNPs with heterozygous rate < 0.25, missing rate < 0.1, and minor allele frequency > 0.1). Association analyses were performed using both a generalized linear model (GLM) and a mixed linear model (MLM) in TASSEL (v5.0), with population structure parameters (principal components and kinship matrix) incorporated as covariates to control for population stratification and improve statistical power. The genome‐wide significance threshold for CTI associations was determined using the conservative Bonferroni correction approach (α = 0.05). LD pruning was performed using PLINK with the parameters “–indep‐pairwise 50 5 0.2” (window size = 50, step size = 5, *r* ^2^ threshold = 0.2), resulting in 111 159 independent SNPs. The corrected significance threshold was therefore calculated as 4.50e‐7. Significant SNP‐trait associations were visualized through Manhattan plots and quantile‐quantile plots generated using the R package “CMplot.” Given the large number of statistical tests in our dataset, even a small FDR would likely yield a large number of false positive SNP associations. We therefore adopted the conservative Bonferroni correction for multiple tests in this study following the common practice in published literature [5, 83].

### 5.6 RNA Extraction and RT‐qPCR Experiments

Total RNA was isolated from *Arabidopsis thaliana* and *Camellia sinensis* tissues using the FastPure Universal Plant Total RNA Isolation Kit (RC411‐01, Vazyme), with RNA purity and concentration verified by a NanoDrop 2000 spectrophotometer (Thermo Fisher Scientific, USA). First‐strand cDNA synthesis was performed with 1 µg total RNA using PrimeScript RT Master Mix (Takara) following the manufacture's protocol, followed by dilution to 150 ng/µL working concentration. RT‐qPCR assay was performed in triplicate biological replicates using Hieff qPCR SYBR Green Master Mix (11201ES03, YEASEN) on a CFX connect Real‐Time PCR system (Bio‐Rad, USA), with melting curve analysis confirmed primer specificity. The reference genes *CsACTIN* (tea plants) and *ACTIN2* (*Arabidopsis*) were used for normalization, with relative expression levels calculated using 2^−ΔCt^ method. All primer sequences used in this study are listed in Data S7.

### 5.7 Plasmid Construction and Genetic Transformation

For overexpression studies, the full‐length coding sequence (CDS) of the target gene was PCR‐amplified and cloned into *pCAMBIA130.5.1‐GFP* (digested with BamH I and Spe I). For virus‐induced gene silencing, around 250‐bp gene‐specific fragments were inserted into *pTRV2* (digested with Xbal and BamH I). Promoter‐GUS fusions were generated by cloning promoters into *pBI121*. For dual‐luciferase reporter assay, transcription factors and promoters were separately inserted into *pGreenII‐62SK* (effector) and *pGreenII‐0800LUC* (reporter). The yeast hybrid assay employed *pGADT7* (AD), *pGBKT7* (BD), *pHIS2.1*, and *pABAI* vectors. For LUC complementation imaging (LCI) assay, the CDSs were inserted into *pCAMBIA1300‐nLUC* and *pCAMBIA1300‐cLUC*, respectively. For bimolecular fluorescence complementation (BiFC) assays, the CDSs were inserted into *pFGC‐YN173* and *pFGC‐YC155*, respectively. All constructs were verified by Sanger sequencing before transformation into respective host systems.

### 5.8 Subcellular Localization Assays

The coding sequences of target genes were fused to GFP in the *pCAMBIA1305.1* vector and transformed into *Agrobacterium* GV3101 (AC1003S, WEIDI). *Nicotiana benthamiana* leaves were co‐infiltrated with the GFP fusion constructs and the nuclear marker DAPI (4′,6‐diamidino‐2‐phenylindole), using an empty vector as a control. After 48 h of incubation at 22°C under 16‐h light/8‐h dark conditions, GFP fluorescence signals were visualized using a confocal laser scanning microscope. Three independent biological replicates were performed for each construct.

### 5.9 Antisense Oligonucleotide (AsODN) Assays

Gene‐specific antisense oligonucleotides (AsODN) were designed using Sfold software (https://sfold.wadsworth.org/cgi‐bin/soligo.pl) targeting specific regions of the coding sequence in corresponding genes. At least three complementary AsODN primers (Data S7) were diluted and mixed to 20 µm/L in sterile water. The “Shuchazao” tea shoots that consist of an apical bud and two adjacent leaves were immersed into AsODN solution for 6–48 h under controlled conditions (25°C, 70% humidity). Silencing efficiency was monitored by RT‐qPCR at post‐treatment 6, 12, 24, 36, and 48 h (n ≥ 3 biological replicates), with the optimal time point selected for subsequent experiments.

### 5.10 Transient Overexpression and Virus‐Induced Gene Silencing Assay in Tea Plants

For overexpression studies, target genes were cloned into *pCAMBIA1305.1‐GFP* and transformed into *Agrobacterium* strain GV3101. Final cultures (OD600 = 1.0) in infiltration buffer (10 mm MES, 10 mm MgCl_2_, 150 µm acetosyringone) were pressure‐infiltrated into young tea leaves using a needleless syringe. For VIGS assays, gene‐specific fragments (∼250 bp) were cloned into *pTRV2* and co‐infiltrated with *pTRV1* (1:1 ratio) carrying the *Agrobacterium* strain GV3101_pSoup19 helper plasmid. Transformation efficient was verified by RT‐qPCR analysis of target gene expression at 1, 6‐, 9‐, 12‐, and 20‐days post‐infiltration (n ≥ 3 biological replicates). To assess potential off‑target effects, we designed two independent VIGS constructs targeting non‑overlapping regions of the target gene. The expression levels of target genes in the tea plants were examined by RT‑qPCR, and there was no significant alteration in the VIGS plants compared with empty vector controls.

### 5.11 Freezing Tolerance Assays

For tea plant cold tolerance evaluation, one‐year‐old “Shuchazao” seedlings were subjected to −5°C for 1 h. Following 30 min dark adaptation, the maximum photosynthetic efficiency of photosystem II (*Fv/Fm*) was measured using an imaging pulse‐amplitude‐modulated (PAM) chlorophyll fluorometer (Heinz Walz, GmbH, Effeltrich, Germany). For *Arabidopsis* cold tolerance evaluation, the transgenic *Arabidopsis* lines (soil‐grown and 1/2 MS cultured) were treated to −10°C (1 h) and −6°C (2 h), respectively. The freezing phenotypes and survival rate were quantified after 5‐day recovery at 22°C. Physiological assessments included: (1) relative ion leakage measured via conductivity meter (OHAUS, USA), (2) lipid peroxidation (MDA contents, BC0020 kit, Solarbio), (3) superoxide dismutase activity (SOD, BC0170 kit, Solarbio), and (4) ROS scavenging capacity visualization through histochemical staining (NBT for O_2_^−^, DAB for H_2_O_2_). Three biological replicates per treatment were flash‐frozen in liquid nitrogen for subsequent analyses.

### 5.12 Transcriptomic Profiling

Publicly available RNA‐seq data (NCBI SRA: SRP061043) were reanalyzed for gene expression under cold stress [84]. The data were provided as clean reads; therefore, no additional adapter trimming or quality filtering was performed. Clean reads were mapped to the “Shuchazao” genome using HISAT2 (v2.2.1) with default parameters [85], and only uniquely mapped reads were retained. Transcript quantification was performed using RSEM (v1.3.3) [86] to calculate the relative expression value (reads per kilobase of transcript per million mapped reads, FPKM) of each gene. Differential expression analysis between the treated group (n = 3) and control (n = 3) samples was performed using DESeq2 (FDR < 0.05, |log2FC| > 1) [87].

### 5.13 Exogenous EGCG/EGC/EC Treatments

One‐year‐old tea seedlings (*Camellia sinensis*) grown under standard greenhouse conditions (25°C, 16 h light/8 h dark, 70% relative humidity) were used for all treatments. Powdered EC, EGC, and EGCG (each with ≥ 98% purity, Yuanye Biotechnology, Shanghai, China) were separately dissolved in deionized water to prepare 1 mm stock solutions. The stock solutions were freshly prepared on each day of treatment. For each compound, the solution was evenly sprayed onto the leaves of the tea seedlings until runoff (approximately 10 mL per seedling). Spraying was performed once daily for 7 consecutive days. Control seedlings were sprayed with an equal volume of deionized water under the same schedule. For each treatment group (control, EC, EGC, EGCG), at least three independent biological replicates (n ≥ 3) were used, with each replicate consisting of one seedling. After the 7‑day treatment period, all seedlings (including controls) were transferred to a temperature‑controlled chamber and exposed to −5°C for 1 h. Following cold exposure, the seedlings were returned to greenhouse conditions for recovery. Physiological assessments were conducted after the cold treatment, following the protocols described in the previous section.

### 5.14 Yeast One‐Hybrid (Y1H) Assays

The full‐length coding sequences of *CsCCR1*, *CsCBF1/4*, *CsLUX*, and *CsKUA1* were cloned into *pGADT7* vector, respectively. Promoters of target genes were inserted into *pHIS2.1*/*pABAI* vector. Using the combination of empty *pGADT7* and bait plasmids as a negative control, the combination of prey and bait plasmids was co‐transformed into a yeast strain with the PEG/LiAc mediated transformation method according to the yeast protocols handbook. The cotransformants were grown on 2‐deficient selective medium (SD‐Leu‐Trp) for 2 d at 29°C. The positive monoclonal clones were inoculated on SD‐His‐Leu‐Trp plates for 5 d at 29°C, and then the growth phenotype of the yeast colonies was photographed.

### 5.15 Electrophoretic Mobility Shift (EMSA) Assays

The coding sequences of *CsCCR1*, *CsCBF1*, and *CsCBF3* were cloned into the *pMAL‐c5x* vector to generate MBP‐tagged fusion proteins. Following transformation into *Escherichia coli* strain BL21 (DE3), protein expression was induced with 0.4 mm IPTG at 16°C for 16 h. Recombinant proteins were purified using Dextran Resin 6FF (C600695, sangon) according to the manufacturer's protocol. Biotin‐labeled DNA probes containing predicted *cis*‐elements were synthesized and incubated with purified proteins. DNA‐protein complex was detected using the Chemiluminescent EMSA Kit (GS009, Beyotime).

### 5.16 GUS Staining and Dual‐luciferase Reporter Assays

For GUS staining analysis, the *CsCHS1* promoter region (∼1.5 kb upstream of the translation start site) was cloned into *pBI121*‐GUS vector and transformed into *Agrobacterium tumefaciens* GV3101. Transgenic tobacco (*Nicotiana tabacum*) plants were generated through leaf transformation and selected on kanamycin‐containing medium. Two‐weeks old transgenic seedlings were subjected to GUS histochemical staining using the GUS Staining Kit (SL7160, Coolaber). For the dual‐LUC assay, the effector (pGreenII‐62SK) and reporter (pGreenII‐0800‐LUC) constructs were co‐transformed into *A. tumefaciens* GV3101 containing the pSoup19 helper plasmid. Bacterial cultures were grown to OD600 ≈ 0.6 in infiltration buffer (10 mm MgCl_2_, 10 mm MES, 100 µm acetosyringone) and pressure‐infiltrated into the abaxial surface of 4‐week‐old *N. benthamiana* leaves using a needleless syringe. After 12 h dark adaptation, plants were maintained under normal growth conditions for 48 h. Dual‐LUC activity of promoters w ere measured by the ratio of LUC/REN value using a Dual Luciferase Reporter Gene Assay Kit (11402ES60, YEASEN) on a chemiluminescence imaging system (Tanon, Shanghai, China), with firefly luciferase (LUC) values normalized to renilla luciferase (REN) internal control.

### 5.17 Yeast Two‐Hybrid (Y2H) Assays

The coding sequences of *CsCBF1‐4* were cloned into the *pGADT7* prey vectors (AD domain), while *CsCCR1* was inserted into the *pGBKT7* bait vector (BD domain). All constructs were verified by Sanger sequencing before co‐transformation into yeast strain Y2HGold using the PEG/LiAc method. Transformants were initially selected on synthetic defined (SD) medium lacking leucine and tryptophan (SD/‐Leu/‐Trp) at 29°C for 2 days. Protein–protein interactions were validated by plating on quadruple dropout medium (SD/‐Leu/‐Trp/‐His/‐Ade) supplemented with 300 ng/mL aureobasidin A. Growth was monitored for 5 days at 29°C, with empty vector combinations serving as negative controls. Three independent colonies were analyzed for each interaction pair to ensure reproducibility.

### 5.18 luciferase Complementation Imaging (LCI) Assays

The *CsCBF1/CsCBF3* were subcloned into *pCAMBIA1300‐nLUC*, while *CsCCR1* was inserted into *pCAMBIA1300‐cLUC* expression vectors. These constructs were transformed into GV3101 cells, followed by co‐infiltration of two different plasmid combinations into 4‐week‐old tabacco leaves using needleless syringes. After 48 h incubation under normal growth conditions (22°C, 16/8 h light/dark), the backs of the leaves were sprayed with 1 mm D‐Luciferin potassium salt and LUC signals were detected using a chemiluminescence imaging system.

### 5.19 Bimolecular Fluorescence Complementation (BiFC) Assays

According to a prior report [51], the coding sequences of *CsCBF1/CsCBF3* were cloned into the pFGC‐YN173 vector, while *CsCCR1* was cloned into the pFGC‐YC155 vector. Following GV3101 transformation of different construct groups, 4‐week‐old tobaccos were used for protein interaction analysis. YFP signals were visualized using a laser‐scanning confocal microscope (Leica TCS SP8, Germany).

### 5.20 H_2_DCFDA Staining Assays

Following a previously described method, intracellular ROS levels were detected using the fluorescent probe H2DCFDA [88]. Briefly, leaves were soaked in staining buffer (10 mm Tris‐HCl, 50 mm KCl, 50 µm H_2_DCFDA, 0.02% Tween‐20 (v/v), pH 7.2) and subjected to vacuum infiltration at −0.6 MPa for 30 min to facilitate dye uptake. Subsequently, the leaves were washed with distilled water to remove any unincorporated dye. Fluorescence signals were then captured and quantified with a laser‐scanning confocal microscope (Leica TCS SP8, Germany).

### 5.21 Statistical Analysis

All raw data were preliminarily sorted, and outliers were assessed before formal analysis. All experiments included at least three independent biological replicates (sample size n ≥ 3) with three technical triplicates. Data are presented as Mean ± standard deviation. Statistical analyses were performed using GraphPad Prism 9.0 with two‐tailed Student's *t*‐test for pairwise comparisons (^*^ *p* < 0.05, ^**^ *p* < 0.01, ^***^ *p* < 0.001, and ^****^ *p* < 0.0001). Multiple group comparisons were conducted via one‐way analysis of variance (one‐way ANOVA), followed by a two‐tailed Fisher's least significant difference (LSD) post hoc test. Value of *p* < 0.05 was considered statistically significant. Correlation coefficient analysis was conducted using the *Pearson* method implemented in *R*.

## Author Contributions

E.X. and W.T. conceived and designed the project; Y.W., W.T., Q.W. and F.L. analyzed the data and performed bioinformatics analysis; Y.W., D.W., Q.W., Q.G. and Y.Z. performed the experiments; Z.Z. and C.S. gave suggestions and discussions; Y.W. draft original manuscript; E.X., W.T. and Y.W. revised the manuscript; W.W. provided the plant materials. All authors have read and approved the final manuscript.

## Conflicts of Interest

The authors declare no competing interests.

## Supporting information




**Supporting File 1**: advs76225‐sup‐0001‐SuppMat.docx.

**Supporting File 2**: advs76225‐sup‐0002‐Data.xlsx.

## Data Availability

Data supporting the findings of this work are available within this paper and its Supplementary files. The DNA sequencing of tea accessions and transcriptome data used in this study can be freely obtained from the National Genomics Data Center (NGDC, https://ngdc.cncb.ac.cn/) database under BioProject number of PRJCA045366. All primers generated in this study are provided in Data S7. Source data are provided with this paper.
