# Supplementary material for: Natural Variation of COLD and CATECHINS REGULATOR 1 Coordinately Fine‐Tunes Cold Tolerance and Tea Quality in Tea Plants
Source: Adv Sci (Weinh). 2026 Jun 22:e76225. Online ahead of print. doi: 10.1002/advs.76225 (PMC13336641; doi:10.1002/advs.76225)
Supplement: Supplementary file 1 — Supporting File 1: advs76225‐sup‐0001‐SuppMat.docx. [file ADVS-9999-e76225-s002.docx]

**Supplementary information**

**Natural variation of COLD AND CATECHINS REGULATOR 1 coordinately fine-tunes cold tolerance and tea quality in tea plants**

Wang *et al.*

**Supplementary Figure 1. Population genetic diversity analysis of 108 tea accessions. (A)** Sequencing statistics showing read depth (x-axis) and mapping rate (y-axis) for each variety; n = 108. **(B)** The density of single-nucleotide polymorphisms (SNPs) across the 15 tea plant chromosomes. **(C)** The distribution of SNPs on 15 chromosomes in all tea varieties. **(D)** Principal component analysis of the population. Province abbreviations: AH (Anhui), FJ (Fujian), ZJ (Zhejiang), GZ (Guizhou), HB (Hubei), HN (Hunan), JX (Jiangxi), JS (Jiangsu), SD (Shandong), SX (Shaanxi), TW (Taiwan), YN (Yunnan).

**Supplementary Figure 2. Cold tolerance diversity in tea population across two consecutive years. (A-E)** Frequency distribution of REC, MDA, Pro, SOD, and CTI in 2023. **(F-J)** Frequency distribution of REC, MDA, Pro, SOD, and CTI in 2024. **(K-N)** Correlation of 4 cold tolerance indicators between 2023 and 2024, with each scatter plot representing one accession. Physiological indicator abbreviations: REC (Relative electrical conductivity), MDA (malondialdehyde), Pro (proline), SOD (superoxide dismutase), and CTI (composite cold tolerance index). R values were generated by Pearson correlation analysis. *P* values were calculated using Student’s *t* test. n = 108.

**Supplementary Figure 3. Frequency distribution and correlation of specific secondary metabolites in tea population in two consecutive years. (A)** Frequency distribution of tea-specific secondary metabolites in 108 tea varieties in 2023. **(B)** Frequency distribution of tea-specific secondary metabolites in 108 tea varieties in 2024. **(C)** The correlation scatter plot of secondary metabolites in tea population between years in 2023 and 2024. EC, epicatechin; ECG, epicatechin gallate; EGC, epigallocatechin; EGCG, epigallocatechin gallate; C, catechin; CG, catechin gallate; GC, gallocatechin; GCG, gallocatechin gallate; CAF, caffeine; THB, theobromine; THP, theophylline. *R* values were generated by Pearson correlation analysis. *P* values were calculated using Student’s *t* test. n = 108.

**Supplementary Figure 4. Heatmap displaying variation and correlation of CTI and secondary metabolites accumulations across all accessions. (A)** Correlation coefficient among tea-specific secondary metabolites in all tea varieties in 2023. **(B)** Correlation coefficient among tea-specific secondary metabolites in all tea varieties in 2024. EC, epicatechin; ECG, epicatechin gallate; EGC, epigallocatechin; EGCG, epigallocatechin gallate; C, catechin; CG, catechin gallate; GC, gallocatechin; GCG, gallocatechin gallate; CAF, caffeine; THB, theobromine; THP, theophylline. Negative correlations are indicated by blue squares, and the positive correlations are in red. R values were generated by Pearson correlation analysis; *P* values were calculated using Student’s *t* test. The red rectangle highlights the top significant correlation between the two metabolites. **(C)** Heatmap displaying best linear unbiased estimates (BLUE) values of CTI and tea-quality-related secondary metabolites accumulation across accessions. Color scale represents normalized (log_2_-transformed) values from low (blue) to high (red). n = 108.

**Supplementary Figure 5. Identification of candidate loci for BLUE values of key secondary metabolites in two consecutive years. (A)** Schematic workflow for identifying genetic loci associated with CTI and secondary metabolites. **(B-I)** Manhattan plots for tea quality related genome-wide association study (GWAS) using BLUE values across two consecutive years. Arrows indicate genes involved in corresponding metabolites. CG: catechin gallate, GC: gallocatechin, GCG: gallocatechin gallate, CAF: caffeine, THB: theobromine, THP: theophylline.

**Supplementary Figure 6. Identification of candidate loci for** **CTI using GWAS.**

**(A-B)** Manhattan plot for CTI in first year with imputed generalized linear model (GLM) and mixed linear model (MLM), respectively. **(C)** Manhattan plot for CTI in second year with three different models. **(D)** GWAS for CTI in two consecutive years with three different models. GLM is in blue dots, MLM is in yellow dots, and purple dots indicate FarmCPU. Color scale bars indicate the density of SNPs.

**Supplementary Figure 7. A single-nucleotide polymorphism in *CsCCR1* coding region was associated with CTI and catechins alteration. (A)** Linkage disequilibrium heatmap spanning *CsCCR1* gene body, 3 kb upstream and downstream regions. **(B)** Comparison of CTI and catechins (EGCG, EGC, EC) levels among *CsCCR1* genotypes CC (n = 85) and AA (n = 5). Data in bar plot represents Mean ± SD. The number above the bar indicates the mean values.

**Supplementary Figure 8. Verification of *CsCCR1*-overexpressing *Arabidopsis* and catechins accumulation under cold stress. (A)** Subcellular localization of CsCCR1. GFP, green fluorescence protein. Bars = 40 μm, n = 3. **(B-C)** Verification of *CsCCR1*-silencing and overexpressing plants. Data represent Mean ± SD, n = 3. Asterisks indicate significant differences relative to control set determined by student’s *t*-tests. (**P* < 0.05, ****P* < 0.001, *****P* < 0.0001). **(D)** The calculated values for linearity (*R^2^* > 0.999). **(E)** Chromatograms and precision (RSD = 1.56%, n = 6) of standards (Retention time: 4.96 (GA), 7.369 (THB), 8.4(GC), 9.857 (THP), 12.555 (EGC), 14.116 (CAF), 14.517 (C), 20.1 (EC), 21.393 (EGCG), 23.946 (GCG), 29.129 (ECG), CG (30.071)). **(F-G)** Differential accumulation of catechin or catechin derivatives among 73 differential accumulation flavonoids (VIP ≥ 1.00, Fold change ≥ 1.45 or ≤ 0.50) between control and cold treated tea shoots. The color scale represents normalized flavonoids content. Triangles represent catechin derivatives, n = 3.

**Supplementary Figure 9. Expression levels of catechins related genes under cold stress. (A)** *CsCHS1***, (B)** *CsFLS1***, (C)** *CsSCPL4***, (D)** *CsSCPL5* and **(E)** *CsELIP1*. CA1-6h: cold acclimated at 10 ℃ for 6 h; CA1-7d: 10/4 ℃ (day/night) for 7 d; CA2-7d: 4/0 ℃ (day/night) for 7 d. All the data are presented as Mean ± SD, n = 3. Statistical significance was determined by student’s *t*-tests (**P* < 0.05, ***P* < 0.01, ****P* < 0.001, *****P* < 0.0001).

**Supplementary Figure 10. Functional characterization of *CsELIP1* in cold tolerance and catechins accumulation. (A-B)** Functional domains and phylogenetic analysis of ELIP in 10 plants. **(C)** Flavonoid contents in *CsELIP1*-silencing and *CsELIP1*-overexpressing tea seedlings. n = 12. **(D)** Catechins accumulations in *CsELIP1*-silencing and *CsELIP1*-overexpressing tea seedlings. n = 3. **(E)** Relative expression of *CsELIP1* in WT and 12 *CsELIP1*-overexpressing *Arabidopsis* lines. n = 3. **(F)** Freezing tolerance in WT and two *CsELIP1*-overexpressing lines. n = 3. The data are presented as Mean ± SD. Asterisks indicate significant differences relative to control set determined by student’s *t*-tests. Significance was determined by student’s *t*-tests (**P* < 0.05, ***P* < 0.01, ****P* < 0.001, ns indicates no significance).

**Supplementary Figure 11. CsCCR1 could not regulate the transcription of *CsDFR1/CsCHS2/CsCHI1/CsLAR1/CsF3Ha/ANS/ANR*. (A)** Dual-LUC assays in tobacco leaves and *Arabidopsis* protoplasts. **(B-C)** Expressions of *ANS* and *ANR* in *CsCCR1*-overexpressing *Arabidopsis* and WT. Data represent Mean ± SD, n = 3. Asterisks indicate significant differences. Significance was determined by student’s *t*-tests. ***P* < 0.01, “ns” indicates no significance).


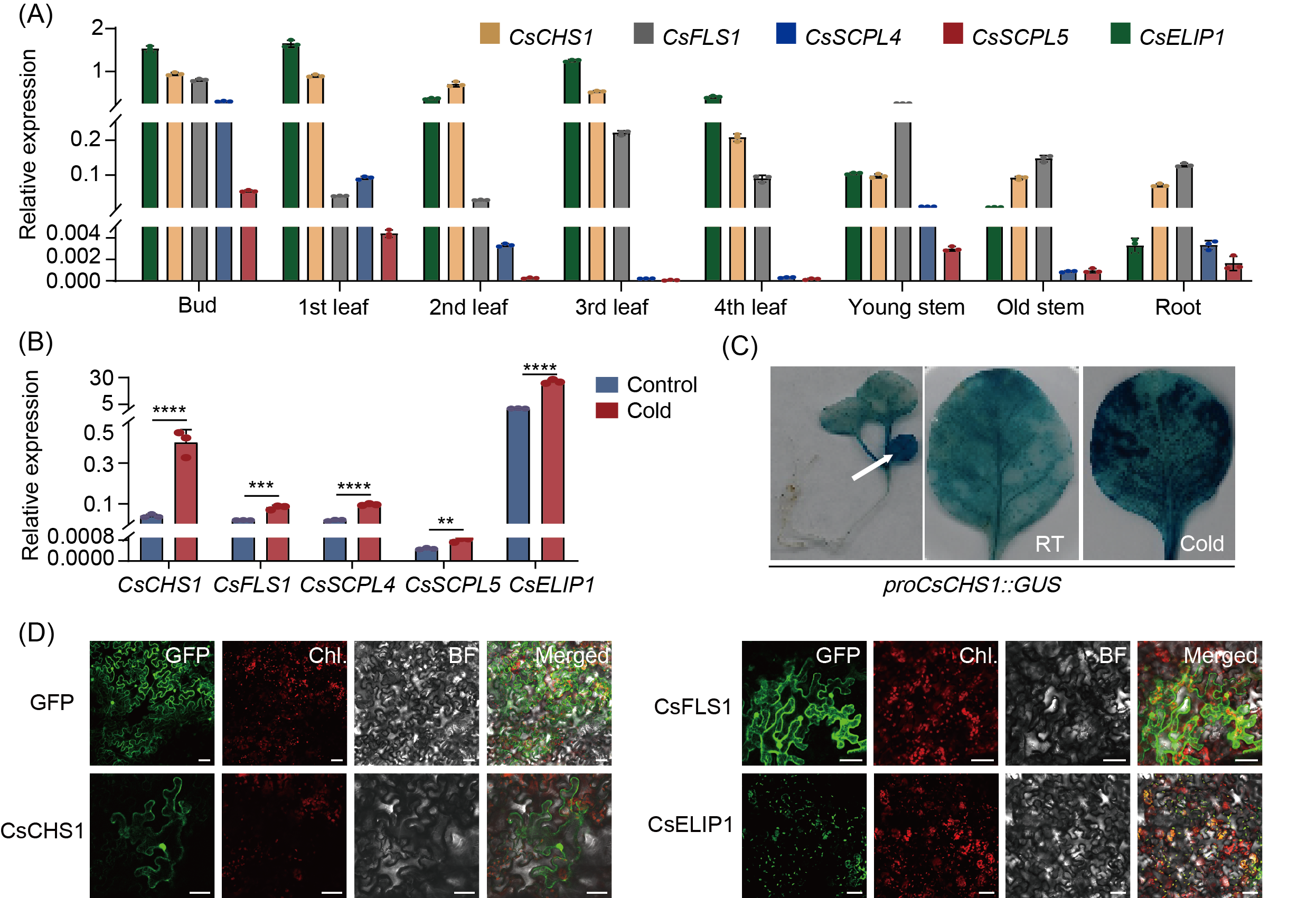


**Supplementary Figure 12. Expression pattern and subcellular localization of CsFLS1, CsELIP1, CsCHS1, CsSCPL4, and CsSCPL5. (A)** High expression of *CsFLS1*, *CsELIP1*, *CsCHS1*, *CsSCPL4*, and *CsSCPL5* in representative tea organs. **(B)** Expression of *CsFLS1*, *CsELIP1*, *CsCHS1*, *CsSCPL4*, and *CsSCPL5* were up-regulated under cold stress. Data represent Mean ± SD, n = 3. Asterisks indicate significant differences. Significance was determined by student’s *t*-tests (***P* < 0.01, ****P* < 0.001). **(C)** GUS staining of *proCsCHS1* showing high expression of *CsCHS1* in young leaves and cold treatment. RT (room temperature). **(D)** Subcellular localization of CsCHS1, CsFLS1 and CsELIP1. GFP, green fluorescence protein. Chl, chloroplast. BF, bright field. Scale bars = 40 μm, n = 3.

**Supplementary Figure 13. Relative expression of *CsCHS1, CsFLS1, CsSCPL4*, *CsSCPL5,* and *CsELIP1* in corresponding gene silencing and overexpressing tea plants. (A)** Expression of *CsCHS1* in silencing and overexpressing tea plants. **(B)** Expression of *CsFLS1* in silencing and overexpressing tea plants. **(C)** Expression of *CsSCPL4* in silencing and overexpressing tea plants. **(D)** Expression of *CsSCPL5* in silencing and overexpressing tea plants. **(E)** Expression of *CsELIP1* in silencing and overexpressing tea plants. Data represent Mean ± SD, n = 3. Significance was determined by student’s *t*-tests (**P* < 0.05, ***P* < 0.01, ****P* < 0.001, *****P* < 0.0001).

**Supplementary Figure 14. Identification of DRE motif on the promoter of *CsCHS1*/*CsFLS1/CsSCPL4/CsSCPL5/CsELIP1*.** DRE: dehydration-responsive element (CCGAC).

**Supplementary Figure 15. Nuclear localization of CsCBF1 and CsCBF3 and cold-induced expression of *CsLUX* and *CsKUA1*.** **(A)** Subcellular location of CsCBF1 and CsCBF3. The *35S:CsCBF1-GFP* vectors and *35S:CsCBF3-GFP* were introduced into tobacco leaves, respectively. GFP, green fluorescence protein. DAPI, nucleus marker. BF, bright field. Scale bars = 40 μm. **(B)** Relative expression of *CsLUX* and *CsKUA1* under cold acclimation. Data was presented in Mean values, n = 3. CA1-6h: cold acclimated at 10 ℃ for 6 h; CA1-7d: 10/4 ℃ (day/night) for 7 d; CA2-7d: 4/0 ℃ (day/night) for 7 d.

**Supplementary Figure 16. The interaction between CsCCR1 and CsCBF1/3 was enhanced by cold stress in tea plants.** **(A)** Bimolecular fluorescence complementation (BiFC) assays confirming that cold treatment enhanced the interaction between CsCCR1 and CsCBF1/3. YFP, yellow fluorescence protein. DAPI, nucleus marker. Scale bars = 40 μm. **(B)** Mean intensity of YFP released by interaction between CsCCR1 and CsCBF1/3. **(C)** LCI assays confirming that cold treatment enhanced the interaction between CsCCR1 and CsCBF1/3. **(D)** Protein intensity of CsCBF1/ CsCBF3/CsCHS1/CsFLS1/CsSCPL4/CsSCPL5/CsELIP1 under cold stress and control set. Data represent means ± SD, n = 3.


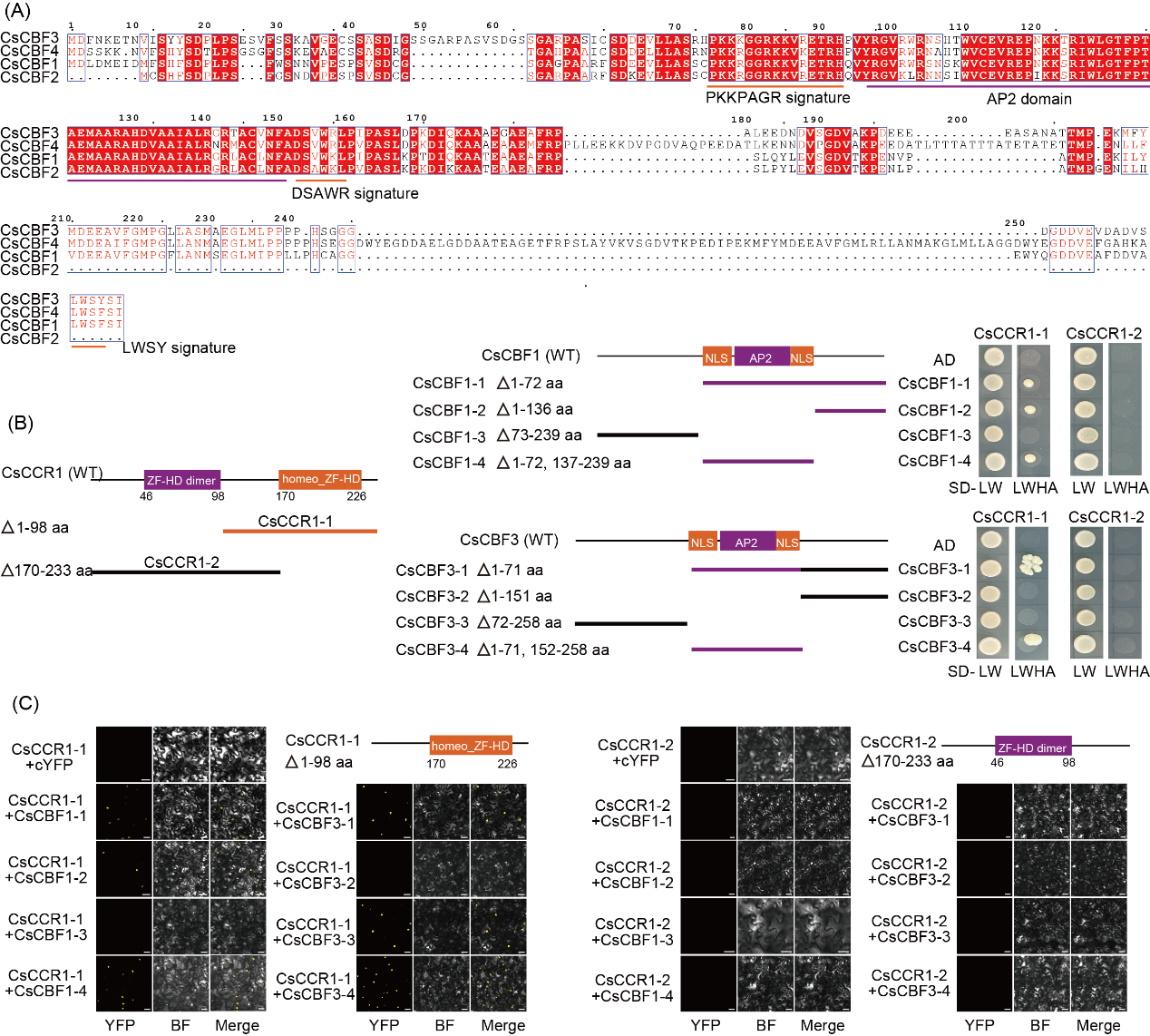


**Supplementary Figure 17. Truncation assays for interaction’s structural residues analysis between CsCCR1 and CsCBF1/3. (A)** Alignment analysis of the amino acid sequences of CsCBFs family. **(B)** Yeast two-hybrid (Y2H) assays showing interaction of C-terminal of CsCCR1 with AP2 domain and C-terminal of CsCBF1, AP2 domain of CsCBF3. **(C)** Bimolecular fluorescence complementation (BiFC) assays confirming interaction of C-terminal of CsCCR1 with AP2 domain and C-terminal of CsCBF1, AP2 domain of CsCBF3. Scale bars = 40 μm, n = 3.

**Supplementary Figure 18.** **Diurnal expression patterns of *CsLUX* and *CsCCR1* and dynamic accumulation of catechins in tea plants.** Diurnal expression patterns and dynamic accumulation of catechins were detected at 4-hour intervals over two consecutive days. RT, normal temperature (control condition); Cold: 4℃ treatment. White bars indicate 12 h light period, while gray bars represent 12 h dark period. ZT, zeitgeber time. Data represent Mean ± SD, n = 3.

**Supplementary Figure 19. Relative expression of *CsCHS1, CsFLS1, CsSCPL4*, *CsSCPL5,* and *CsELIP1* and catechins accumulation in *CsLUX*-silencing and *CsLUX*-overexpressing tea plants.** EC, epicatechin; ECG, epicatechin gallate; EGC, epigallocatechin; EGCG, epigallocatechin gallate. Data represent Mean ± SD, n = 3. Significance was determined by student’s *t*-tests (**P* < 0.05, ***P* < 0.01, ****P* < 0.001, *****P* < 0.0001). “ns” indicates no significance.

**Supplementary Figure 20. Relative expression of *CsCHS1, CsFLS1, CsSCPL4*, *CsSCPL5,* and *CsELIP1* and catechins accumulation in *CsKUA1*-overexpressing tea plants. (A)** Expression level of *CsKUA1* in eight tea organs. Bud (B), leaf (L), young stem (YS), old stem (OS), root (R). **(B)** Relative expression of *CsCHS1, CsFLS1, CsSCPL4*, *CsSCPL5,* and *CsELIP1* and catechins accumulation in *CsKUA1*-overexpressing tea plants and control. **(C)** Individual flavan-3-ol levels (EGCG, EGC, EC, ECG) in *CsLUX*-silenced and *CsLUX*-overexpressing (OE) tea plants compared to controls. Data represent Mean ± SD, n = 3. Significance was determined by student’s *t*-tests (**P* < 0.05, ***P* < 0.01, ****P* < 0.001, *****P* < 0.0001). “ns” indicates no significance.

**Supplementary Figure 21. Overexpressing *CsCCR1* enhanced ROS scavenging ability in *Arabidopsis*.** **(A)** Go terms of 787 differentially expressed genes (DEGs) in wild-type and *CsCCR1*-overexpressing *Arabidopsis* using Gene Ontology (GO) annotations. The DEGs were filtered with *P* < 0.05 and fold change >2. **(B)** Expression levels (FPKM) of *PRX9* (*PEROXIDASE 9*), *PRX40* (*PEROXIDASE 40*), *GPX4* (*glutathione peroxidase 4*), *MSRB6* (*methionine sulfoxide reductase B6*). Data represent Mean ± SD, n = 3. Significance was determined by student’s *t*-tests (**P* < 0.05, ***P* < 0.01, ****P* < 0.001).

**
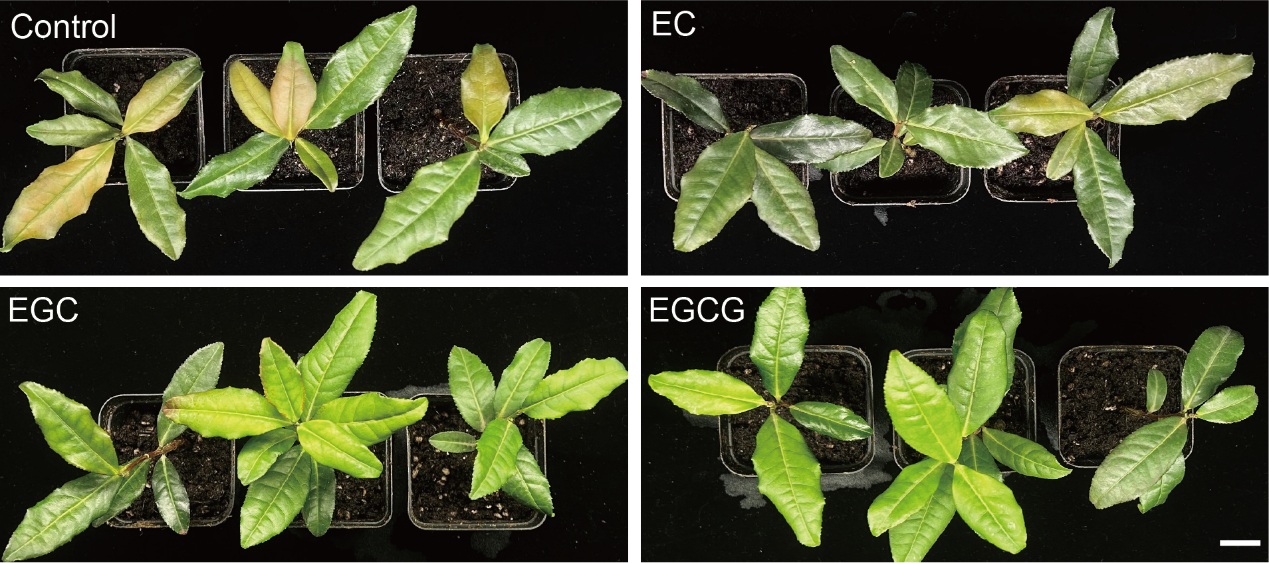
**

**Supplementary Figure 22. Freezing phenotypes after spraying 1 mM EC, EGC, EGCG and control set.** EC, epicatechin; EGC, epigallocatechin; EGCG, epigallocatechin gallate. Scale bar = 2 cm.

**Supplementary Figure 23. Enzymatic antioxidants (SOD, POD, CAT, APX) and non-enzymatic antioxidants (GSH and AsA) in silencing, overexpressing catechins-biosynthetic genes, and catechin-treated plants. (A)** Catechins biosynthetic genes silenced samples. **(B)** Catechins biosynthetic gene overexpressed samples. **(C)** Catechin-treated tea plants. Data represent Mean ± SD, n = 3. Significance was determined by student’s *t*-tests (**P* < 0.05, ***P* < 0.01, ****P* < 0.001, *****P* < 0.0001). “ns” indicates no significance.

**Supplementary Figure 24. Effects of missense mutation (A356C) on cold tolerance and catechins. (A)** *Fv/Fm* in OE-*CsCCR1^A^*, OE-*CsCCR1^C^* and control lines. **(B)** Individual catechins accumulation in OE-*CsCCR1^A^*, OE-*CsCCR1^C^* and control lines. FW: fresh weight. EC, epicatechin; ECG, epicatechin; EGC, epigallocatechin; EGCG, epigallocatechin gallate. Data represent Mean ± SD, n = 3.


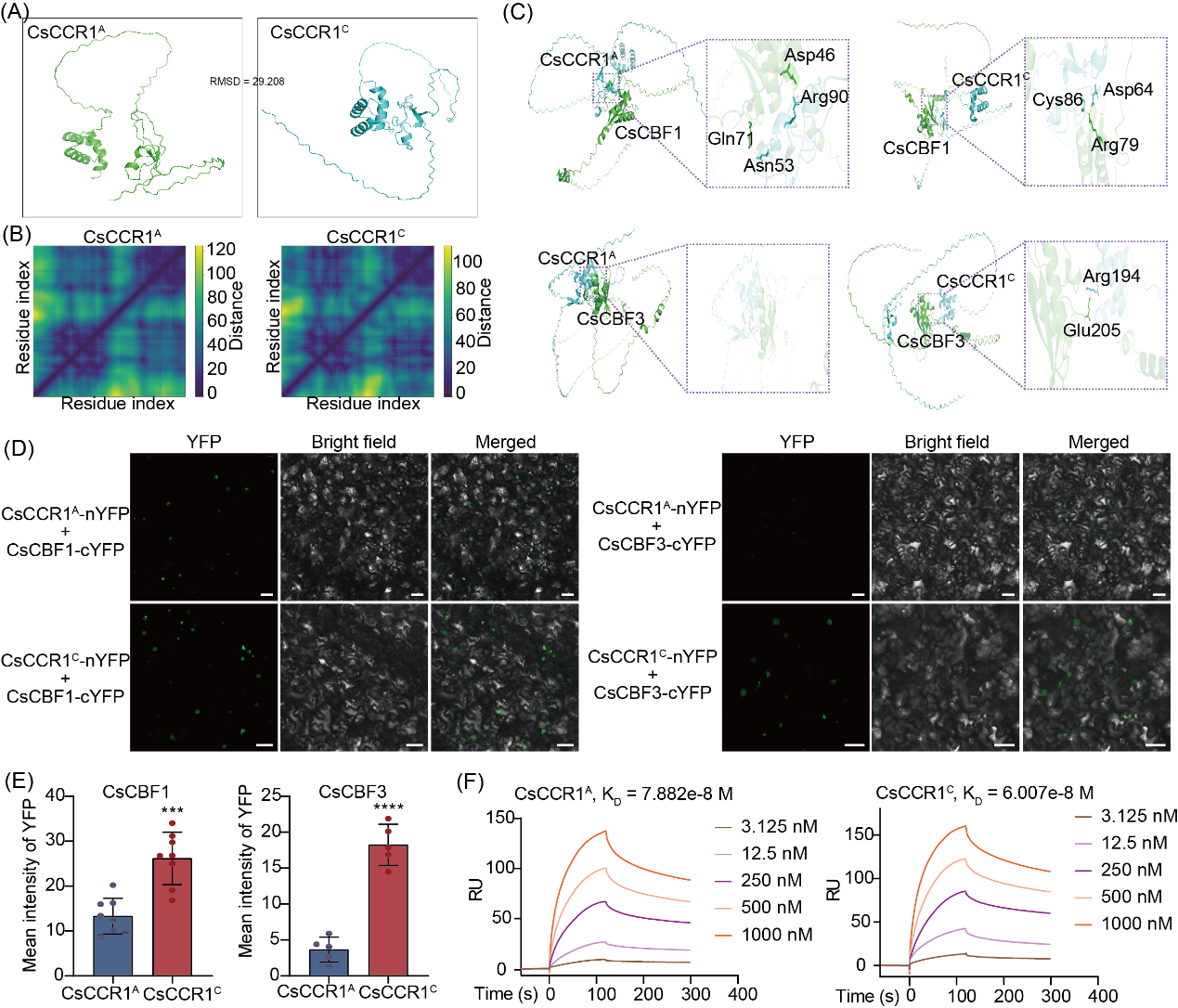


**Supplementary Figure 25. Effects of missense mutation (A356C) on interaction between CsCCR1 and CsCBF1/3. (A)** Protein structure of CsCCR1^A^ and CsCCR1^C^. **(B)** Distances between amino acid residues in CsCCR1^A^ and CsCCR1^C^. **(C)** Molecular docking between CsCBF1/3 and CsCCR1. **(D-E)** BiFC assays showing the binding differences between CsCCR1^A^ and CsCCR1^C^. YFP, yellow fluorescence protein. Scale bars = 40 μm. **(F)** SPR assays showing the binding differences between CsCCR1^A^ and CsCCR1^C^. Data represent Mean ± SD, n = 3. Significance was determined by student’s *t*-tests (***P* < 0.01, ****P* < 0.001, *****P* < 0.0001).
